# Gene duplications and gene loss in the epidermal differentiation complex during the evolutionary land-to-water transition of cetaceans

**DOI:** 10.1038/s41598-021-91863-3

**Published:** 2021-06-10

**Authors:** Karin Brigit Holthaus, Julia Lachner, Bettina Ebner, Erwin Tschachler, Leopold Eckhart

**Affiliations:** Skin Biology Laboratory, Department of Dermatology, Medical University of Vienna, Vienna, Austria

**Keywords:** Differentiation, Evolutionary biology, Evolutionary genetics, Marine biology

## Abstract

Major protein components of the mammalian skin barrier are encoded by genes clustered in the Epidermal Differentiation Complex (EDC). The skin of cetaceans, i.e. whales, porpoises and dolphins, differs histologically from that of terrestrial mammals. However, the genetic regulation of their epidermal barrier is only incompletely known. Here, we investigated the EDC of cetaceans by comparative genomics. We found that important epidermal cornification proteins, such as loricrin and involucrin are conserved and subtypes of small proline-rich proteins (SPRRs) are even expanded in numbers in cetaceans. By contrast, keratinocyte proline rich protein (KPRP), skin-specific protein 32 (XP32) and late-cornified envelope (LCE) genes with the notable exception of LCE7A have been lost in cetaceans. Genes encoding proline rich 9 (PRR9) and late cornified envelope like proline rich 1 (LELP1) have degenerated in subgroups of cetaceans. These data suggest that the evolution of an aquatic lifestyle was accompanied by amplification of SPRR genes and loss of specific other epidermal differentiation genes in the phylogenetic lineage leading to cetaceans.

## Introduction

The skin of terrestrial tetrapods, i.e. reptiles, birds and mammals, has evolved to protect against mechanical stress and loss of water in a dry environment^1,2^. The barrier function of the skin is mediated by the epidermis and depends on the differentiation of keratinocytes^3,4^. The basal layer of the epidermis contains stem cells and proliferating cells whereas suprabasal layers consist of post-mitotic, differentiating cells. The latter activate a specific gene expression program that leads to accumulation of keratin intermediate filaments, strengthening of intercellular junctions and cross-linking of proteins to form so-called cornified envelopes or corneocytes^4,5^. Tight junctions and extracellular lipids complete the barrier in terrestrial mammals^6,7^.

Cetaceans have evolved from terrestrial and amphibious ancestors by adapting to a fully aquatic lifestyle^8,9^. The integument has undergone notable changes including the loss of pelage, a pronounced thickening of the epidermis and thickening of the hypodermal fat layer (blubber). The adaptations of the skin were driven by modifications and loss of genes^10–14^. As expected, the loss of hair and nails was associated with the loss of keratins and keratin-associated proteins that are expressed specifically in these skin appendages of terrestrial mammals^13,14^. Similarly, the alteration of keratinocyte differentiation in the epidermis, characterized by the accumulation of many cell layers, absence of a granular layer and retentiation of nuclei in the outermost layer, commonly referred to as parakeratosis, is the consequence of changes of epidermis-related genes. Examples include the loss of keratins *KRT1*, *KRT2* and *KRT10*^13^, *DSG4*, *DSC1*, *TGM5*, *ALOXE3*^12^, *PSORS1C2*^15^, *GSDMA* and *IL37*^16^, and *CASP14*^17^. However, a major cluster of epidermal differentiation genes, known as the Epidermal Differentiation Complex (EDC)^18–20^, has not been fully characterized in cetaceans, perhaps due to the peculiar structure of the genes in the EDC. Among the proteins encoded by EDC genes, only S100As and S100 fused-type proteins (SFTPs) have a protein fold that can be predicted from their amino acid sequence. By contrast, so-called simple EDC (SEDC) genes, characterized by the presence of the entire coding sequence in the second of two exons, encode proteins of highly repetitive sequences that are enriched in few amino acids. For example, loricrin is extremely rich in glycine and serine and keratinocyte proline-rich protein (KPRP) is rich in proline. SFTP genes were reported to be lost, with the notable exception of *FLG* (filaggrin) in dolphins^17^, whereas only partial accounts of SEDC genes of cetaceans are available^21,22^.

Here, we performed comparative genomics to determine the gene composition of the EDC in cetaceans. Based on the identification of SEDC genes in phylogenetically diverse cetaceans and their next terrestrial relatives, we propose a model for the differential evolution of EDC genes in cetaceans.

## Results

### Identification of the epidermal differentiation complex (EDC) in cetaceans

**Figure 1:**
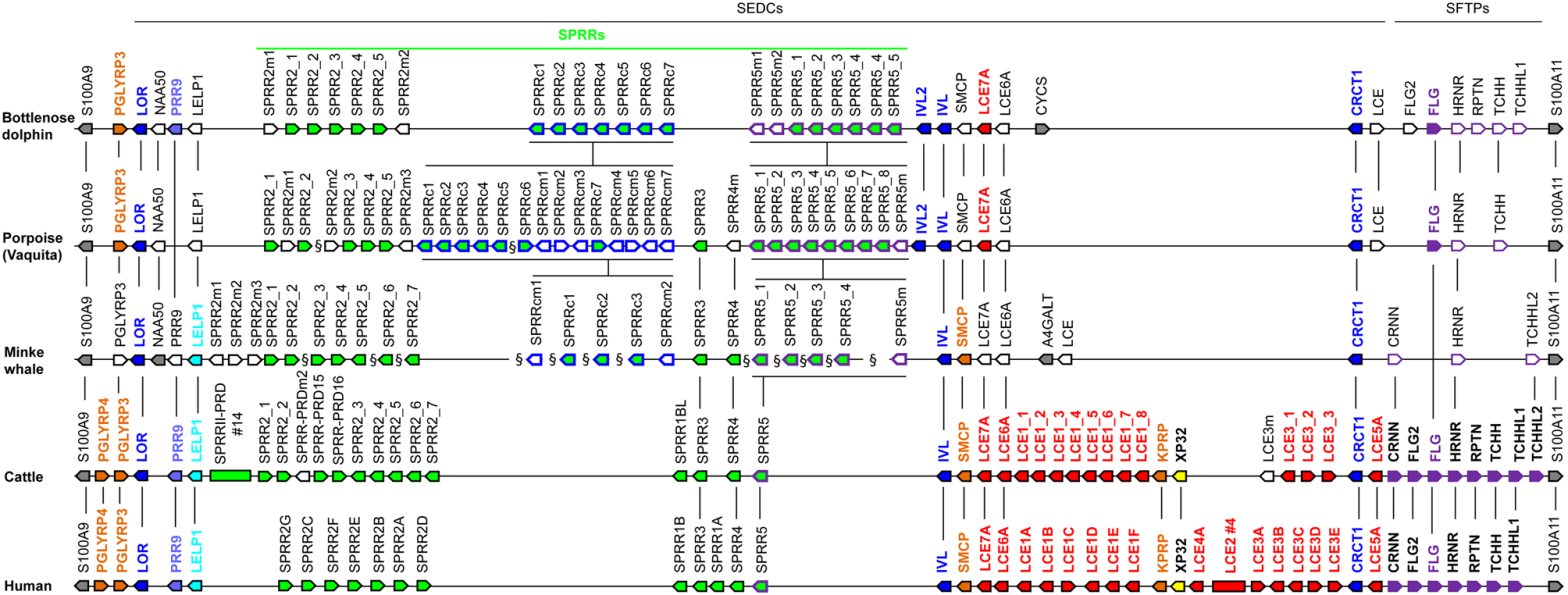
Comparison of the EDC in cetaceans and terrestrial mammals. EDC genes in the region from *S100A9* to *S100A11* are schematically depicted by arrows pointing in the direction of transcription. Vertical lines indicate orthology of genes and gene families in different species. The colors of gene arrows mark gene families. White arrows indicate genes in which the coding sequence is disrupted by premature stop codons or frameshifts. Several clusters of similar genes are depicted by rectangles with the number of genes being indicated after the symbol “#”. Due to the yet incomplete genome sequence assemblies of the minke whale and vaquita (porpoise), there are gaps (§) in their EDC and real discontinuity cannot be excluded. The arrangements of genes in minke whale and vaquita (porpoise) are based on the model of synteny with the dolphin EDC. The pseudogenes *NAA50*, *A4GALT* and *CYCS* lack orthologs in the human and bovine EDCs. Species: bottlenose dolphin (*Tursiops truncatus*), vaquita (*Phocoena sinus*), minke whale (*Balaenoptera acutorostrata scammoni*), cattle (*Bos taurus*), and human (*Homo sapiens*). SEDC, simple EDC gene (1 coding exon); SFTP, S100 fused-type protein.

We investigated the EDC in species of cetaceans for which whole genome sequences had been published (Supplementary Tables S1). The study was focused on bottlenose dolphin (*Tursiops truncatus*)^23^, vaquita (*Phocoena sinus*)^24^, and minke whale (*Balaenoptera acutorostrata scammoni*)^25^ (Supplementary Tables S2-S4; Supplementary Fig. S1-S3). These species belong to the families of oceanic dolphins (Delphinidae), porpoises (Phocoenidae), and rorquals (Balaenopteridae), respectively. Dolphins and porpoises are toothed whales (Odontoceti) whereas rorquals are baleen whales (Mysticeti). The complete EDC was also analyzed in the genome of the blue whale (*Balaenoptera musculus*) (Supplementary Table S5). The arrangement of genes in the EDC was compared to that in cattle (Supplementary Table S6; Supplementary Fig. S4), a fully terrestrial relative of cetaceans within the taxonomic order Cetartiodactyla, and human. A continuous sequence of the EDC locus was available for the dolphin whereas a preliminary map of the EDC including sequence gaps or positions of ambiguous sequence assembly was drawn for vaquity (porpoise) and minke whale (Fig. 1).

In all species investigated, the highest number of EDC genes belongs to the SEDC type, characterized by the presence of the entire coding sequence within one exon^26^. *PGLYRP3* and *FLG*, belonging to the SFTP-type of EDC genes, are located on the borders of the SEDC gene cluster in the dolphin and porpoise whereas, in agreement with previous reports^11,17^ both *PGLYRP3* and *FLG* are absent in the minke whale (Fig. 1) and the blue whale (Supplementary Table S5). The PGLYRP3 protein of the bottlenose dolphin and vaquita is truncated relative to its orthologs and contains only one instead of two PGRP domains (Supplementary Fig. S1, S2, S4).

Among SEDC genes, two of the best characterized cornification genes of terrestrial mammals, i.e. *LOR* (loricrin) and *IVL* (involucrin)^5^, are conserved in cetaceans (Fig. 1), with *IVL* being present in the form of two gene copies in the bottlenose dolphin and the vaquita (Fig. 1; Supplementary Fig. S5). Likewise, *CRCT1* (cysteine rich C-terminal 1) was conserved in all cetaceans investigated (Fig. 1; Supplementary Fig. S6). *PRR9*, *LELP1*, *SMCP* (sperm mitochondria associated cysteine rich protein) showed differential conservation (Supplementary Fig. S7) with *PRR9* being intact (devoid of premature in-frame stop codons or frameshift mutations) in the dolphin (*T. truncatus*) and the North Pacific right whale (*Eubalaena japonica*) (Supplementary Fig. S7A), but not in the vaquita, minke and blue whales (Supplementary Tables S3-S5). *LELP1* is intact in the minke whale whereas it contains a frameshift mutation in dolphin and vaquita (Fig. 1; Supplementary Fig. S7B). *SMCP* which is an atypical EDC gene because of its expression exclusively in the testis^27–29^, is conserved in minke whale and in sperm whale but not in dolphin and porpoise (Supplementary Fig. S7C). *SPRR* and *LCE* gene families are also differentially conserved in cetaceans, as will be described in detail further below. *XP32* (Skin-specific protein 32), also referred to as *C1ORF68* (chromosome 1 open reading frame 68) in humans, and *KPRP* have been lost in cetaceans (Fig. 1). *KPRP* encodes a truncated protein in the amphibious relative of cetaceans, the hippopotamus (Suppl. Fig. S8), but a recent study estimated that *KPRP* was intact in the last common ancestor of Whippomorpha (cetaceans and hippopotamuses)^30,31^.

### Evidence for SEDC gene expression in the skin

**Figure 2:**
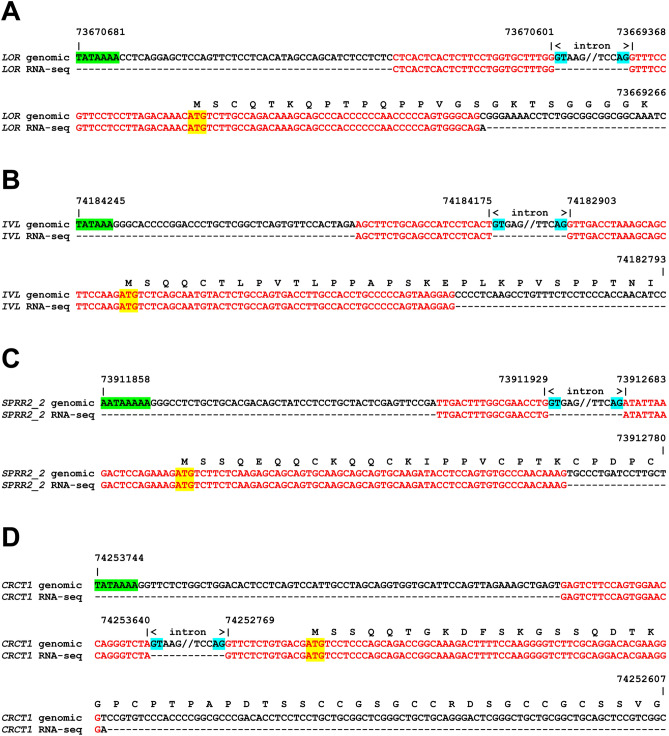
Evidence for SEDC gene expression in dolphin skin. The skin transciptome of the bottlenose dolphin (*Tursiops truncatus*) (sequence read archive, accession number SRX2398721) was screened for transcripts encoding SEDC proteins. Amino acid sequences of SEDC-encoded proteins LOR (**A**), IVL (**B**), SPRR2_2 (**C**) and CRCT1 (**D**) were used as queries in tBLASTn searches. RNA-seq reads covering the start of the coding sequence and the intron-spanning 5’-untranslated region are shown. The complete sequence of the RNA-seq reads and the corresponding genomic sequence including flanking regions are shown. Introns are indicated by 5 nucleotides on the border to the flanking exons and a symbol (//) to indicate a gap in the aligned sequence. The translation of the coding sequence is shown above the nucleotide sequence. The start codon, the TATA box and splice site signals (GT and AG) are highlighted in yellow, green and blue shading, respectively. Nucleotide positions in the genomic DNA (GenBank accession number NC_045763.1) are indicated. Red fonts indicate sequence identity.

To test whether SEDC genes are expressed in the skin of cetaceans and to localize the borders of exons, we performed tBLASTn searches for the predicted mRNAs in the skin transcriptome database of the dolphin^32^ and aligned mRNA reads to the genome sequence. Expression of loricrin, involucrin, *SPRR2* and *CRCT1* was confirmed by the identification of intron-spanning sequence reads (Fig. 2). The intron-spanning reads confirmed the 2-exon structure of these SEDC genes in cetaceans. In addition, mRNAs corresponding to parts of exon 2 of several other EDC genes were found in the dolphin skin transcriptome (Supplementary Table S2).

### Subtypes of SPRRs have expanded in copy numbers in cetaceans

In all three cetaceans analyzed in this study SPRRs formed the largest gene family within the EDC (Fig. 1). Although the precise arrangement and number of SPRR genes in cetaceans is currently unknown because of incomplete or ambiguous assembly of the genome sequence in this region (Supplementary Tables S2-S5), we could identify a higher number of SPRR genes in cetaceans (n = 16–21) than in the human EDC (n = 12). The numbers of cetacean *SPRR*s is close to that of the ruminants such as the cattle. However, rumen-specific type-II small proline-rich proteins with paired (PRD) repeats (SPRRII-PRDs)^33,34^ are absent in cetaceans.

**Figure 3:**
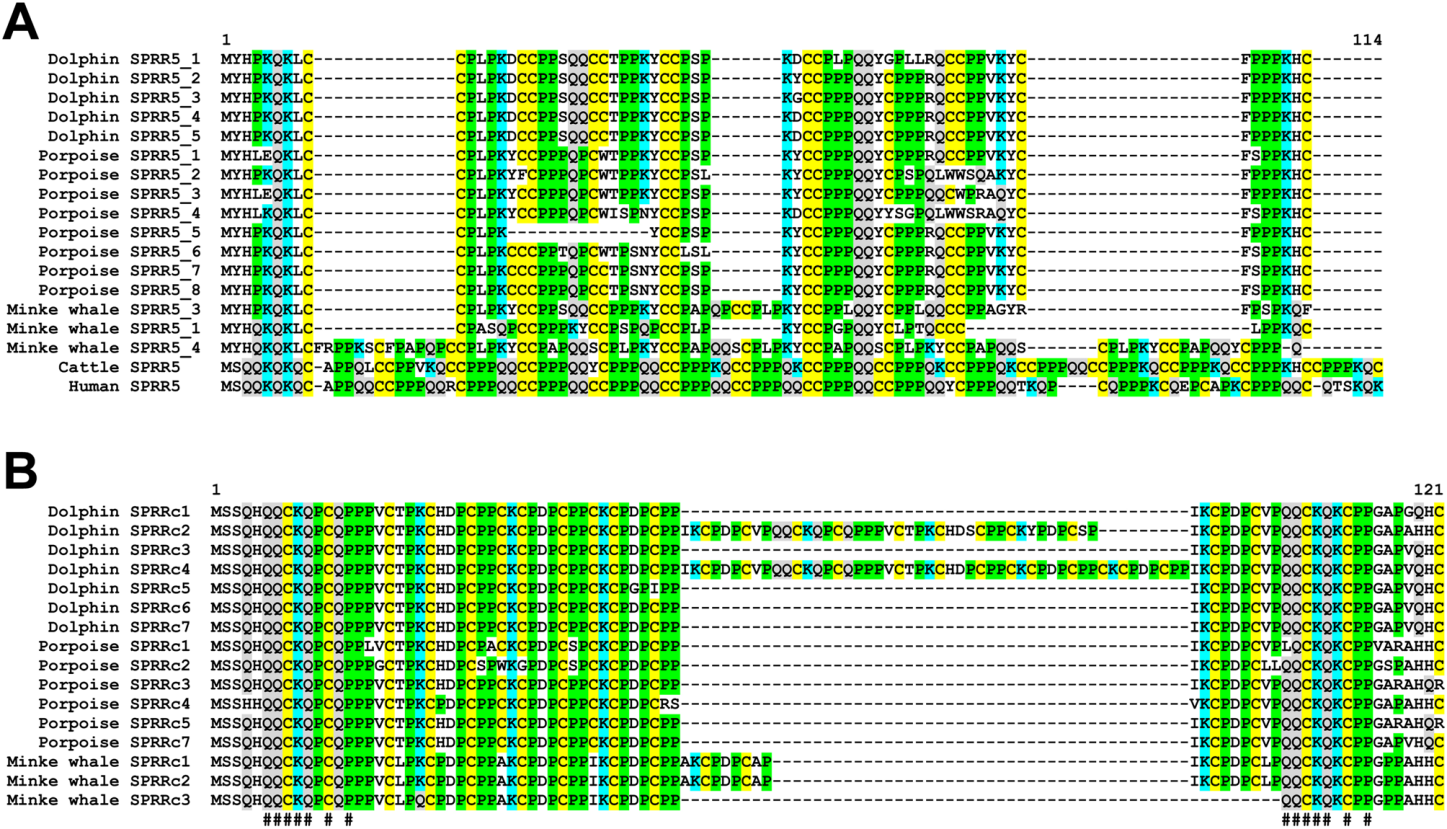
Small proline rich 5 (SPRR5) and cetacean-specific SPRR (SPRRc) are present in high copy numbers in cetaceans. (**A**) Amino acid sequence alignment of SPRR5 proteins of bottlenose dolphin, porpoise and minke whale, cattle and human. In cetaceans SPRR5 is expanded in copy number whereas only one gene is present in cattle and human. SPRR5 has distinctive cysteine (C) duplets. Cetacean SPRR5s are rich in aromatic acid residues compared to cattle and human. (**B**) Amino acid alignment of SPRRs belonging to a cetacean-specific subtype (SPRRc). The presence of a sequence motif, that is evolutionarily conserved in many SEDC proteins of amniotes, at 2 positions of SPRRc amino acid sequences is indicated with “#” below the sequences. Cysteine (C), glutamine (Q), lysine (K) and proline (P) are highlighted by yellow, grey, blue and green shading. Information about the genes encoding the proteins is provided in Supplementary Tables S2-S6. Species: cattle (*Bos taurus*), dolphin (*Tursiops truncatus*), human (*Homo sapiens*), minke whale (*Balaenoptera acutorostrata scammoni*), porpoise (*Phocoena sinus*).

Two subtypes of SPRR genes are uniquely expanded in cetaceans. First, the EDCs of cetaceans contain 4–8 copies of *SPRR5* which is a single-copy gene in humans and cattle (Fig. 3). SPRR5 is the only SPRR with duplets of cysteine residues (Fig. 3A, Supplementary Fig. S9). The current genome sequence assembly of the bottlenose dolphin contains a cluster of at least 8 further *SPRR5* genes, flanked by sequence gaps on chromosome 10 (Supplementary Fig. S10), but their annotation and location outside of the EDC (chromosome 1) is considered uncertain. Second, SPRR genes, tentatively termed SPRR-cetacean type (SPRRc), are present between *SPRR2* and *SPRR3* in the EDC of cetaceans but not in the EDC of cattle and human (Fig. 3B). At least three SPRRc copies are present in the minke whale and seven in bottlenose dolphin and vaquita (Fig. 1). SPRRc proteins contain 2 copies of the sequence motif, QQCKQXCXP (IUPAC-IUB code)^35^, which is very similar to an evolutionarily ancient motif at the amino-terminus of diverse SEDC proteins^26,36^ (Fig. 3B).

### LCE genes with the exception of LCE7A have been lost in cetaceans

**Figure 4:**
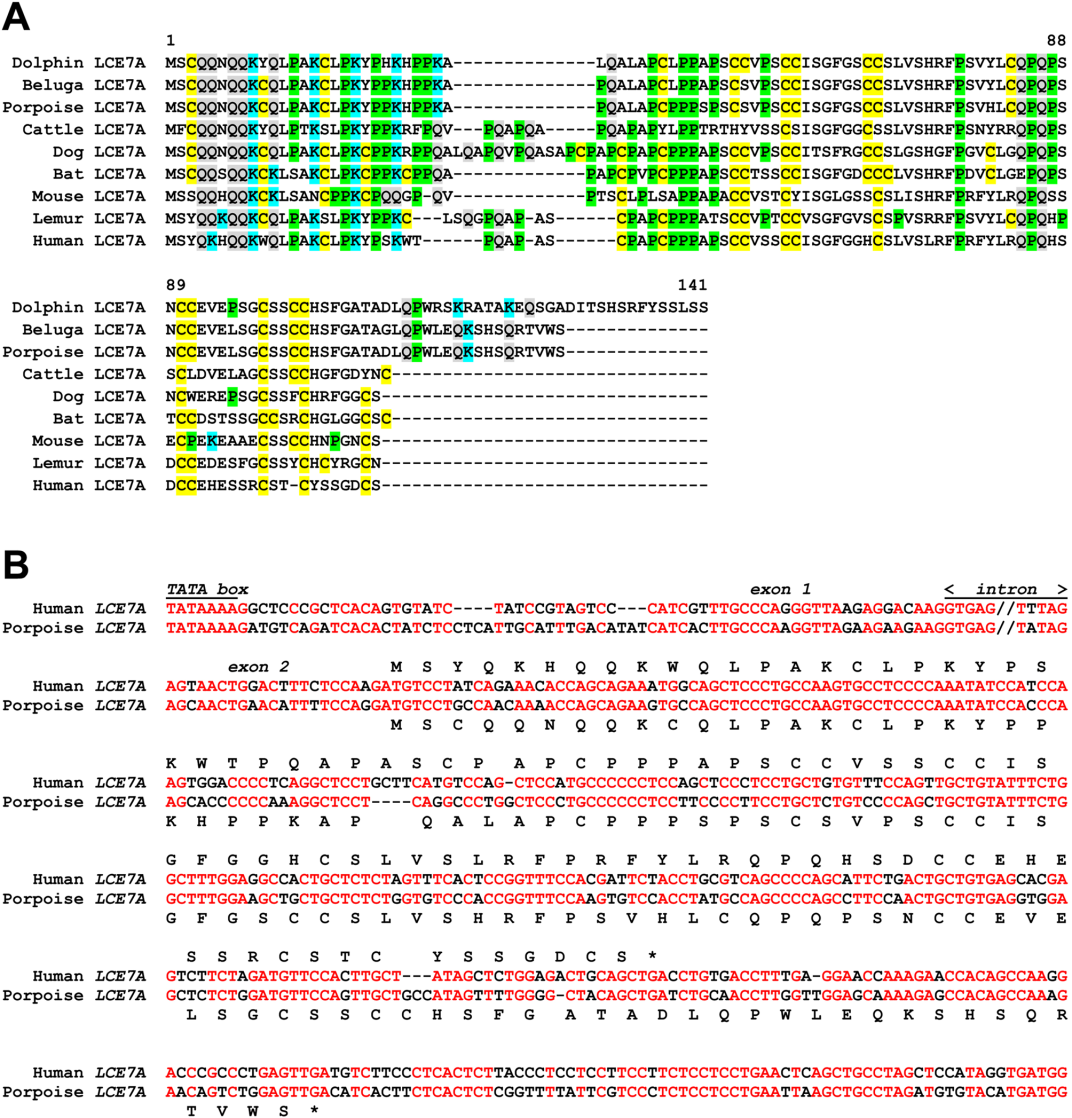
LCE7A is conserved in dolphin and porpoise and expressed in human epidermal keratinocytes. (**A**) Amino acid sequence alignment of LCE7A proteins of dolphin (*Tursiops truncatus*, Suppl. Figure S1), porpoise (*Phocoena sinus*, Suppl. Figure S2), beluga (*Delphinapterus leucas*, ENSDLEP00000014034), cattle (*Bos taurus*, XP_024845590.1), sheep (*Ovis aries*, XP_027832872.1), dog (*Canis lupus familiaris*, ENSCAFP00000035891), bat (*Rhinolophus ferrumequinum*, ENSRFEP00010020125), mouse (*Mus musculus*, ENSMUSP00000141278), lemur (*Prolemur simus*, ENSPSMP00000000419), human (*Homo sapiens*, ENSG00000285946). Cysteine (C), glutamine (Q), lysine (K) and proline (P) are highlighted by yellow, grey, blue and green shading. (**B**) Nucleotide sequence alignment of human and porpoise (*Phocoena sinus*) *LCE7A* genes. Introns are indicated by 5 nucleotides on the border to the flanking exons and the symbol “//” indicates a gap in the aligned sequence. The translation of the coding sequence is shown above the nucleotide sequence. Asterisks indicate the end of the proteins due to a stop codon. Red fonts indicate sequence identity. The human sequence corresponds to GenBank accession number NC_000001.11, nucleotides 152859963–152861111, excluding gap as indicated).

Besides SPRR genes, LCEs form a second large gene family in the EDC of cattle and human. By contrast, only a single LCE gene was identified in dolphin and porpoise and no LCE is present in the EDC of the minke whale (Fig. 1). Because of shared synteny and best reciprocal similarity scores, this gene was identified as ortholog of *LCE7A* which was previously identified in sheep^34^. Comparative genomics showed that *LCE7A* is conserved in the blue whale (Supplementary Table S5), the beluga whale and phylogenetically diverse mammals including humans (Fig. 4A). We identified *LCE7A* in the human EDC as an uncharacterized gene that was recently annotated in the ENSEMBL database (accession number ENSG00000285946). Comparison of nucleotide sequences of human and porpoise *LCE7A* showed conservation of the TATA box in the promoter, splice sites at the borders of the two exons and most of the coding sequence with the exception of the 3’-end where a frameshift leads to an elongation of the coding sequence in the porpoise (Fig. 4B). The expression of human *LCE7A* was confirmed by RT-PCR analysis of epidermal keratinocytes and the sequence was submitted to GenBank (accession number MW556765).

## Discussion

**Figure 5:**
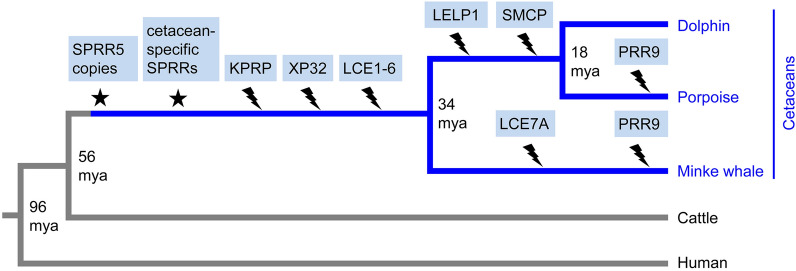
Schematic model of EDC gene evolution in cetaceans. Gene duplications leading to the origin of new genes (star) and gene loss event (flash symbol) are indicated on a cladogram that shows the relation of species investigated in this study. Gene origin and loss were inferred from the distribution of genes in extant species. Evolutionary divergence times (millon years ago, mya) are indicated at the divergence points.

The results of this study demonstrate that the EDC of cetaceans differs significantly from that of terrestrial mammals, suggesting that the evolution of the fully aquatic lifestyle of cetaceans was accompanied by major changes in the differentiation program of epidermal keratinocytes. The EDC contributes to protective functions of the epidermis by controlling the protein composition of the mechanically resistant keratinocytes on the skin surface and by contributing to the antimicrobial defense of the skin. Genes encoding barrier proteins such as *LOR*, *IVL*, *SPRR*s and *CRCT1* have been conserved whereas others, such as *KPRP*, *XP32* and most *LCE*s have been lost in cetaceans and yet others, such as *PRR9* and *LELP1* have been lost in subclades of cetaceans (Fig. 5). Important amino acid sequence features of EDC proteins, such as high contents of either glycine (in LOR), glutamine (in IVL), cysteine (in CRCT1) and proline (in SPRRs) are conserved in cetaceans, indicating that major mechanisms of skin barrier maturation have been retained during evolutionary adaptation to aquatic life. Of note, glutamine (Q), which is targeted by transglutamination during cornification, is enriched to higher levels in minke whale IVL (> 40% Q) than in any terrestrial mammal investigated (Supplementary Fig. S5).

The number of SPRRs has increased during the evolution of cetaceans, suggesting that they play special roles in whales, porpoises and dolphins. Human and mouse SPRRs are components of corneocytes and also act as scavengers of reactive oxygen species^37,38^. They are predominantly expressed in hyperproliferative states such as wound healing^39^. Interestingly, the epidermis of cetaceans shares some features with the hyperproliferative epidermis of terrestrial mammals including molecular markers such as keratin K6^13,40^. We put forward the hypothesis that the evolution of the thick epidermis of cetaceans was associated with the constitutive expression of *SPRR*s possibly with particular roles of the two subtypes of *SPRR* that have expanded in numbers in cetaceans.

One of the most striking differences between the EDC in cetaceans and terrestrial mammals is a massive loss of *LCE* genes which form a large subcluster of the EDC in cattle and humans. LCE proteins accumulate by transcriptional upregulation when keratinocytes are exposed to ultraviolet radiation^41^ and other types of stress^39^, and function as structural components of corneocytes^42^ and antimicrobial proteins^43^. In this regard it is interesting that other EDC genes with antimicrobial functions such as *PGLYRP4*^11^ and *HRNR*^44^ have also been lost in cetaceans. An as-yet poorly characterized *LCE* gene, *LCE7A*^34^ is conserved in some but not all cetaceans and, as we show here, also in the human EDC. Thus, the results of this study indicate that most *LCE* genes are dispensable in the aquatic environment of cetaceans and provide the basis for further investigations of *LCE7A* in both cetaceans and humans.

The evolutionary changes in the EDC must be evaluated in the context of changes at other genome loci, especially those involved in the regulation of the skin barrier function. It was already reported that *CASP14*, *TGM5*, *DSG4* and *DSC1* were lost in cetaceans^12,17^. In addition, many genes involved in the control of skin immune responses have been lost in cetaceans, e.g. CCL27, *IL20*, *IL36A*, *IL36B*, *IL37*, *IL38*, *NLRP10*, *PYDC1* and *PSORS1C2*^15,16,45,46^. It is thus possible that not only the functions of individual genes but the function of entire gene interaction networks related to cutaneous protection and defense have declined in cetaceans. Projects aimed at closing gaps in genome sequences and determining tissue transcriptomes will help to perform comprehensive analyses of the EDC and other epidermal differentiation-associated genes of cetaceans. Furthermore, it will be interesting to investigate the implications of skin adaptations in the resistance to shear stress associated with moving through the aquatic environment, wound healing and defense against microbes.

In conclusion, the EDC has undergone multiple changes during the evolution of cetaceans, indicating that the molecular composition of the epidermis is adapted to aquatic life. The functional characterization of individual epidermal differentiation genes in their normal cellular environment is not possible at present, but may be facilitated by the establishment of in vitro culture and manipulation protocols for keratinocytes of cetaceans in the future^47^.

## Methods

### Ethics statement

The Ethics Committee at the Medical University of Vienna approved the use of skin samples for the isolation and culture of human cells (EK2011/1149). All donors provided written informed consent. All methods were performed in accordance with the relevant guidelines and regulations. Genomes and transcriptomes of cetaceans were investigated exclusively using sequences available in public databases.

### Identification of EDC genes in cetaceans

EDC genes of cetaceans were identified by comparative analysis of the region between S100A9 and S100A11 genes in the genomes of cetaceans (Supplementary Tables S2–S5; Supplementary Fig. S1–S3). Orthology of genes was determined using the criteria of best reciprocal Basic Local Alignment Search Tool (BLAST) hits of the encoded protein sequences and gene locus synteny. Several EDC genes were annotated in the genome sequence assemblies available in the NCBI GenBank and additional genes were identified by iterative tBLASTn searches in which amino acid sequences of newly identified cetacean EDC proteins were used as tBLASTn query sequences in the reference genome sequences and unassembled whole genome shotgun sequences (WGS) of other cetacean species. For species in which an alignment of transcriptome data to genome sequences were available in the section “Genomic regions, transcripts, and products” at https://www.ncbi.nlm.nih.gov/gene/, EDC sequence regions with evidence for transcription were scrutinized for their potential to encode proteins with typical sequence features^26^. Of note, several EDC genes encoding proteins with low sequence complexity were previously annotated as long-noncoding RNAs (lncRNAs) in GenBank. Other EDC genes had been predicted with incorrect borders of exons in GenBank and these prediction were corrected according to orthology with verified EDC genes of other species.

### Comparison of sequences and mapping of gene gain and loss on phylogenetic trees

Amino acid sequences and nucleotide sequences were aligned with MultAlin with manual adjustment^48^. The amino acid composition of proteins was calculated with ProtParam^49^. The domain structure of proteins was analyzed with the NCBI tool for search of conserved domains at https://www.ncbi.nlm.nih.gov/Structure/cdd/wrpsb.cgi^50^. Origin and loss of genes during evolution were inferred from the distribution of orthologous genes in extant species according to the criterion of maximum parsimony. Evolutionary relations and estimated divergence times of the species investigated were obtained from http://www.timetree.org/^51^, last accessed on 20 February 2021.

### RT-PCR amplification of LCE7A in human keratinocytes

Keratinocytes and fibroblasts were isolated and cultured from human skin according to a published protocol^52^. Keratinocytes were induced to differentiate by seeding on top of a collagen matrix containing fibroblasts and subsequent air exposure of the 3D skin models according to a published protocol^52^. RNA was prepared from the keratinocytes in the epidermal compartment of 3D cultures using TriFast (VWR), reverse-transcribed with the Iscript™ kit (Biorad) and subjected to PCR with intron-spanning primers specific for LCE7A (LCE7-s1, 5’-TTGCCCAGGGTTAAGAGGACA-3’ and LCE7-a1, 5’-CTTTGGTTCCTCAAAGGTCAC-3’). The PCR product was purified with the Wizard® SV Gel and PCR Clean-Up System (Promega) and sequenced (Microsynth, Vienna, Austria).

## Supplementary Information

Supplementary Information.

## Data Availability

All data generated or analysed during this study are included in this published article and its Supplementary Information files. The sequence of human LCE7A is available in GenBank at accession number MW556765.
