# Supplementary material for: Gene duplications and gene loss in the epidermal differentiation complex during the evolutionary land-to-water transition of cetaceans
Source: Sci Rep. 2021 Jun 10;11:12334. doi: 10.1038/s41598-021-91863-3 (PMC8192740; doi:10.1038/s41598-021-91863-3)
Supplement: Supplementary file 1 — Supplementary Information. [file 41598_2021_91863_MOESM1_ESM.pdf]

Supplementary Data: Supplementary Tables and Figures

Gene duplications and gene loss in the epidermal differentiation complex during the evolutionary land-to-water transition of cetaceans

Karin Brigit Holthaus, Julia Lachner, Bettina Ebner, Erwin Tschachler, Leopold Eckhart

Content

Supplementary Tables S1-S6

Supplementary Figures S1-S10

Supplementary Table S1. Cetacean species and genome assemblies investigated

Species	Common name	Taxonomy	GenBank assembly accession number	Scope of analysis in this study
Tursiops truncatus	Bottlenose dolphin	Odontoceti	GCF_011762595.1	Complete EDC
Phocoena sinus	Vaquita (porpoise)	Odontoceti	GCF_008692025.1	Complete EDC
Balaenoptera acutorostrata scammoni	Minke whale	Mysticeti	GCF_000493695.1	Complete EDC
Balaenoptera musculus	Blue whale	Mysticeti	GCF_009873245.2	Complete EDC
Delphinapterus leucas	Beluga whale	Odontoceti	GCA_002288925.3	Specific EDC genes
Globicephala melas	Long-finned pilot whale	Odontoceti	GCA_006547405.1	Specific EDC genes
Lagenorhynchus obliquidens	Pacific white-sided dolphin	Odontoceti	GCA_003676395.1	Specific EDC genes
Lipotes vexillifer	Yangtze river dolphin (Baiji)	Odontoceti	GCA_000442215.1	Specific EDC genes
Monodon monoceros	Narwhal	Odontoceti	GCA_005190385.2	Specific EDC genes
Neophocaena asiaeorientalis	Yangtze finless porpoise	Odontoceti	GCA_003031525.1	Specific EDC genes
Orcinus orca	Killer whale	Odontoceti	GCA_000331955.2	Specific EDC genes
Physeter catodon	Sperm whale	Odontoceti	GCA_002837175.2	Specific EDC genes
Sousa chinensis	Indo-pacific humpbacked dolphin	Odontoceti	GCA_003521335.2	Specific EDC genes
Balaenoptera bonaerensis	Antarctic minke whale	Mysticeti	GCA_000978805.1	Specific EDC genes
Balaenoptera physalus	Fin whale	Mysticeti	GCA_008795845.1	Specific EDC genes
Eschrichtus robustus	Grey whale	Mysticeti	GCA_002189225.1	Specific EDC genes
Eubalaena japonica	North Pacific Right whale	Mysticeti	GCA_004363455.1	Specific EDC genes
Megaptera novaeangliae	Humpback whale	Mysticeti	GCA_004329385.1	Specific EDC genes

Note: The complete EDC was investigated in 4 species (Suppl. Tables S2-S5) whereas only a subset of EDC genes were investigated in other species of cetaceans as described in the main text.

Supplementary Table S2. Bottlenose dolphin (*Tursiops truncatus*) EDC genes

Gene	Accession nr.	Gene (CDS) start*	Gene (CDS) end*	Note	RNA-seq reads**	Same CDS prediction in GenBank?	GenBank Gene ID***	GenBank gene description
S100A9	NC_047034.1	73617292	73615022	gene intact	yes	yes	101335451	
PGLYRP3	NC_047034.1	73629575	73636819	gene intact	no	no	101327680	CDS disrupted by mutation
LOR	NC_047034.1	73669342	73668914	gene intact	yes	yes	109547412	
NAA50m	NC_047034.1	73708998	73708489	pseudogene	yes	n.a.	101328349	
PRR9	NC_047034.1	73729110	73728760	gene intact	no	yes	101335475	
LELP1m	NC_047034.1	73759499	73759239	pseudogene	no	no	101315852	CDS disrupted by mutation
SPRR2m1	NC_047034.1	73816225	73816575	pseudogene	yes	no	101334320	CDS disrupted by mutation
SPRR2_1	NC_047034.1	73905710	73905898	gene intact	yes	no	117312700	lncRNA
SPRR2_2	NC_047034.1	73912702	73912890	gene intact	yes	no	117312701	lncRNA
SPRR2_3	NC_047034.1	73919730	73919918	gene intact	yes	no	117312702	lncRNA
SPRR2_4	NC_047034.1	73926723	73926911	gene intact	yes	no	117312703	lncRNA
SPRR2_5	NC_047034.1	73933959	73934147	gene intact	yes	no	109547408	lncRNA
SPRR2m2	NC_047034.1	73976183	73975869	pseudogene	yes	no	109547418	lncRNA
SPRRc1	NC_047034.1	73983454	73983239	gene intact	yes	no	109547418	lncRNA
SPRRc2	NC_047034.1	73990520	73990182	gene intact	yes	no		
SPRRc3	NC_047034.1	73997515	73997300	gene intact	yes	no	117312708	lncRNA
SPRRc4	NC_047034.1	74004641	74004276	gene intact	yes	no	117312704	lncRNA
SPRRc5	NC_047034.1	74011671	74011456	gene intact	yes	no	117312704	lncRNA
SPRRc6	NC_047034.1	74018665	74018450	gene intact	yes	no	117312711	lncRNA
SPRRc7	NC_047034.1	74025652	74025437	gene intact	yes	no		
SPRR5m1	NC_047034.1	74067945	74067770	pseudogene	no	no		
SPRR5m2	NC_047034.1	74077168	74077004	pseudogene	no	no		
SPRR5_1	NC_047034.1	74091193	74090996	gene intact	yes	no	117312721	lncRNA
SPRR5_2	NC_047034.1	74100228	74100031	gene intact	yes	no	117312712	lncRNA
SPRR5_3	NC_047034.1	74109249	74109052	gene intact	yes	no		
SPRR5_4	NC_047034.1	74117394	74117197	gene intact	yes	no	117312719	lncRNA
SPRR5_5	NC_047034.1	74126407	74126210	gene intact	yes	no	117312722	lncRNA
IVL2	NC_047034.1	74170602	74170081	gene intact	yes	no	117313366	different exons
IVL	NC_047034.1	74182880	74181387	gene intact	yes	yes	101337469	
SMCPm	NC_047034.1	74199010	74198759	pseudogene	no	no		
LCE7A	NC_047034.1	74208497	74208114	gene intact	yes	no	109547395	lncRNA
LCE6Am	NC_047034.1	74224167	74223930	pseudogene	yes	no		
Cytochrome c-like	NC_047034.1	74244620	74244940	gene intact	yes	yes	101325461	pseudogene?
CRCT1	NC_047034.1	74252756	74252490	gene intact	yes	no	117312714	lncRNA
LCEm	NC_047034.1	74276761	74276898	pseudogene	yes	no		
FLG	NC_047034.1	74386756	74390861	gene intact	yes	no	117313377	different exons
S100A11	NC_047034.1	74497180	74502108	gene intact	yes	yes	101317964	

Notes: The list includes PGLYRP, SEDC, SFTP genes and the S100A genes, that flank PGLYRP3 or the SFTP region, but not other S100As.

*CDS (coding sequence) is shown for intact genes. For genes that carry inactivating mutations (labeled with "m"), nucleotide positions indicate the region of sequence similarity that was identified by tBLASTn search using orthologous proteins of other species as queries.

**, RNA-seq peaks in the "Genomic regions, transcripts and products" view at www.ncbi.nlm.nih.gov (accessed on 14 July 2020).

***, GenBank gene prediction at the locus of gene predicted in the present study

n.a., not applicable

Supplementary Table S3. Vaquita porpoise (*Phocoena sinus*) EDC genes

Gene	Accession nr.	Gene (CDS) start*	Gene (CDS) end*	Note	RNA-seq reads**	Same CDS prediction in GenBank?	GenBank Gene ID***	GenBank gene description
S100A9	NC_045763.1	112399221	112401464	gene intact	yes	yes	116739746	
PGLYRP3	NC_045763.1	112386941	112379500	gene intact	yes	no	116763406	CDS disrupted
LOR	NC_045763.1	112351297	112351725	gene intact	no			
NAA50m	NC_045763.1	112311293	112311802	pseudogene	no	n.a.	116742596	
LELP1m	NC_045763.1	112291455	112291721	pseudogene	no	no	116748958	CDS disrupted
SPRR2_1	NC_045763.1	112238368	112238102	gene intact	no			
SPRR2m1	NC_045763.1	112219950	112219867	pseudogene	no			
SPRR2_2	NC_045763.1	112164713	112164525	gene intact	yes	no	116765079	lncRNA
SPRR2m2	VOSV01002060.1	6219	6332	pseudogene	n.a.			
SPRR2_3	VOSV01002060.1	13160	13348	gene intact	n.a.			
SPRR2_4	VOSV01002060.1	20226	20411	gene intact	n.a.			
SPRR2_5	VOSV01002060.1	27193	27381	gene intact	n.a.			
SPRR2m3	VOSV01002060.1	41782	41970	pseudogene	n.a.			
SPRR2_6	VOSV01002060.1	102852	102640	gene intact	n.a.			
SPRRc1	VOSV01002060.1	111262	111047	gene intact	n.a.			
SPRRc2	VOSV01002060.1	118245	118030	gene intact	n.a.			
SPRRc3	VOSV01002060.1	123078	122863	gene intact	n.a.			
SPRRc4	VOSV01002060.1	130044	129829	gene intact	n.a.			
SPRRc5	VOSV01002060.1	137012	136797	gene intact	n.a.			
SPRRc6	NC_045763.1	111881958	>111882038	incomplete	no			
SPRRcm1	NC_045763.1	111876807	111876628	pseudogene	no			
SPRRcm2	NC_045763.1	111874990	111875226	pseudogene	no			
SPRRcm3	NC_045763.1	111869818	111869639	pseudogene	no			
SPRRc7	NC_045763.1	111868000	111868215	gene intact	no			
SPRRcm4	NC_045763.1	111842572	111842393	pseudogene	no			
SPRRcm5	NC_045763.1	111840732	111840959	pseudogene	no			
SPRRcm6	NC_045763.1	111835593	111835414	pseudogene	no			
SPRRcm7	NC_045763.1	111828704	111828525	pseudogene	no			
SPPR3	NC_045763.1	111807471	111807812	gene intact	no	yes	116762845	
SPRR4m	NC_045763.1	111789536	111789784	pseudogene	no	yes	116752774	
SPRR5_1	NC_045763.1	111746244	111746441	gene intact	no			
SPRR5_2	NC_045763.1	111736657	111736854	gene intact	no			
SPRR5_3	NC_045763.1	111728232	111728429	gene intact	no			
SPRR5_4	NC_045763.1	111718639	111718836	gene intact	no			
SPRR5_5	NC_045763.1	111704404	111704559	gene intact	no			
SPRR5_6	NC_045763.1	111695740	111695937	gene intact	no			
SPRR5_7	NC_045763.1	111687044	111687241	gene intact	no			
SPRR5_8	NC_045763.1	111679131	111679328	gene intact	no			
SPRR5m	NC_045763.1	111667357	111667554	pseudogene	no			
IVL2	NC_045763.1	111624044	111625180	gene intact	yes	no	116740382	Different CDS
IVL	NC_045763.1	111606516	111607910	gene intact	yes	no	116741544	Different CDS
SMCPm	NC_045763.1	111582493	111582744	pseudogene	no			
LCE7A	NC_045763.1	111572925	111573266	gene intact	no			
LCE6Am	NC_045763.1	111557250	111557396	pseudogene	yes			
CRTC1	NC_045763.1	111521266	111521529	gene intact	no			
LCEm	NC_045763.1	111497572	111497435	pseudogene	no			
FLG	NC_045763.1	111401470	111398297	gene intact	no			
S100A11	NC_045763.1	111298640	111293709	gene intact	yes	yes	116743531	

Notes: The list includes PGLYRP, SEDC, SFTP genes and the S100A genes, that flank PGLYRP3 or the SFTP region, but no other S100As.

The symbols < and > indicate that ends of the coding sequence are not present on the scaffold.

*CDS (coding sequence) is shown for intact genes. For genes that carry inactivating mutations (labeled with "m"), nucleotide positions indicate the region of sequence similarity that was identified by tBLASTn search using orthologous proteins of other species as queries.

**, RNA-seq peaks in the "Genomic regions, transcripts and products" view at www.ncbi.nlm.nih.gov (accessed on 14 July 2020).

***, GenBank gene prediction at the locus of gene predicted in the present study

n.a., not applicable

Supplementary Table S4. Minke whale (*Balaenoptera acutorostrata scammoni*) EDC genes

Gene	Accession nr.	Gene (CDS) start*	Gene (CDS) end*	Note	RNA-seq reads**	Same CDS prediction in GenBank?	GenBank Gene ID***	GenBank gene description
S100A9	NW_006726510.1	1965999	1960001	gene intact	yes	yes	103015302	
PGLYRP3m	NW_006726510.1	1967384	1975102	pseudogene	no	yes	102997690	
LOR	NW_006726510.1	2010474	2009249	incomplete	no			
NAA50	NW_006726510.1	2041771	2041278	gene intact	yes	yes	103015576	
PRR9m	NW_006726510.1	2062506	2062156	pseudogene	no	yes	103015854	
LELP1	NW_006726510.1	2079261	2078962	gene intact	no	yes	103016143	
SPRRm1	NW_006726510.1	2112969	2112805	pseudogene	no			
SPRRm2	NW_006726510.1	2152471	2152767	pseudogene	no			
SPRRm3	NW_006726510.1	2181025	2181211	pseudogene	no			
SPRR2_1	NW_006726510.1	2241586	2241798	gene intact	no			
SPRR2_2	NW_006726510.1	2247783	2247971	gene intact	yes			
SPRR2_3	ATDI01095618.1	1777	1589	gene intact	n.a.			
SPRR2_4	NW_006725822.1	1148	1330	gene intact	yes	yes	114236529	
SPRR2_5	NW_006730590.1	13258	13446	gene intact	n.a.	yes	114238887	
SPRR2_6	NW_006730590.1	20156	20371	gene intact	n.a.	yes	103010144	
SPRR2_7	NW_006730590.1	50938	50735	gene intact	n.a.			
SPRRcm1	NW_006726884.1	1232	1107	pseudogene	n.a.			
SPRRc1	NW_006727216.1	118	32	gene intact	n.a.			
SPRRc2	NW_006727250.1	131	373	gene intact	n.a.			
SPRRc3	NW_006730346.1	57356	57544	gene intact	n.a.			
SPRRcm2	NW_006730346.1	50399	50536	pseudogene	n.a.			
SPRR3	NW_006730346.1	34266	34607	gene intact	n.a.	yes	103001060	
SPRR4	NW_006730346.1	7861	8097	gene intact	n.a.	yes	114238840	
SPRR5_1	NW_006727748.1	452	279	gene intact	n.a.			
SPRR5_2	NW_006726735.1	1106	>1240	incomplete	n.a.			
SPRR5_3	NW_006725396.1	9482	9700	gene intact	n.a.			
SPRR5_4	NW_006726894.1	605	312	gene intact	n.a.			
SPRRm	NW_006726465.1	2611880	2612029	pseudogene	no			
IVL	NW_006726465.1	2563852	2565606	gene intact	no	no	103014087	different exons
SMCP	NW_006726465.1	2547617	2547916	gene intact	no			
LCE7Am	NW_006726465.1	2538143	2538427	pseudogene	no			
LCE6Am	NW_006726465.1	2521055	2521294	pseudogene	no	yes	103013545	
A4GALT	NW_006726465.1	2511160	2512224	gene intact	yes	yes	103013262	
LCEm	NW_006726465.1	2506586	2506850	pseudogene	no			
CRTC1	NW_006726465.1	2475012	2475278	gene intact	no			
S100A11	NW_006726465.1	2248437	2243551	gene intact	yes	yes	103003394	

Notes: The list includes PGLYRP, SEDC, SFTP genes and the S100A genes, that flank PGLYRP3 or the SFTP region, but no other S100As.
Genes on the scaffold with Acc. Nr. NW_006726465.1 were preceded by a gap and genes not homologous to typical EDC genes, indicating either an assembly error or a gene rearrangement.
*CDS (coding sequence) is shown for intact genes. For genes that carry inactivating mutations (labeled with "m"), nucleotide positions indicate the region of sequence similarity that was identified by tBLASTn search using orthologous proteins of other species as queries.
**, RNA-seq peaks in the "Genomic regions, transcripts and products" view at www.ncbi.nlm.nih.gov (accessed on 14 July 2020).
***, GenBank gene prediction at the locus of gene predicted in the present study
n.a., not applicable

Supplementary Table S5. Blue whale (*Balaenoptera musculus*) EDC genes

Gene	Accession nr.	Gene (CDS) start*	Gene (CDS) end*	Note	RNA-seq reads**	Same CDS prediction in GenBank?	GenBank Gene ID***	GenBank gene description
S100A9	NC_045785.1	111417815	111420091	gene intact	yes	yes	118886102	
PGLYRP3m	NC_045785.1	*111412706*	*111407023*	pseudogene	no	yes	118902430	
LOR	NC_045785.1	111373383	111373814	gene intact	yes	yes	118904258	
NAA50	NC_045785.1	111335937	111336446	gene intact	yes	yes	118882364	
PRR9m	NC_045785.1	*111315567*	*111315920*	pseudogene	no	yes	118902377	
LELP1	NC_045785.1	111293987	111294286	gene intact	no	yes	118890599	
SPRR2	NC_045785.1	111225980	111225714	gene intact	no	yes	118894406	
(assembly gap)	*NC_045785.1*	*110793828*	*111166015*	*estimated length 372188*	*n.a.*	*n.a.*		
SPRRc1	NC_045785.1	110790207	110790422	gene intact	yes	no	118905549	lncRNA
SPRRc2	NC_045785.1	110783235	110783450	gene intact	yes	no	118905560	lncRNA
SPRR3	NC_045785.1	110766944	110767381	gene intact	yes	yes	118903907	
SPRR4	NC_045785.1	110748903	110749145	gene intact	no	yes	118895012	
SPRR5_1	NC_045785.1	110712686	110712925	gene intact	no	no		
SPRR5_2	NC_045785.1	110708672	110708932	gene intact	yes	no		
(assembly gap)	*NC_045785.1*	*110439743*	*110705588*	*estimated length 265846*	*n.a.*	*n.a.*		
SPRR5_3	NC_045785.1	110439636	>110439743	incomplete	yes	no		
IVL	NC_045785.1	110404134	110405909	gene intact	yes	yes	118882629	
SMCP	NC_045785.1	110388024	110388323	gene intact	no	no		
LCE7A	NC_045785.1	110378541	110378786	gene intact	no	no		
LCE6Am	NC_045785.1	*110362893*	*110363132*	pseudogene	yes	yes	118890197	
A4GALT-X1	NC_045785.1	110319239	110320612	gene intact	yes	yes	118901765	
CRTC1	NC_045785.1	110283425	110283664	gene intact	yes	no		
CRNNm	NC_045785.1	*110217662*	*110214547*	pseudogene	no	no	118890238	different exons
(assembly gap)	*NC_045785.1*	*110175593*	*110188112*	*estimated length 12520*	*n.a.*	*n.a.*		
S100A11	NC_045785.1	110050348	110045455	gene intact	yes	yes	118892635	

Notes: The list includes PGLYRP, SEDC, SFTP genes and the S100A genes, that flank PGLYRP3 or the SFTP region, but no other S100As.

*, CDS (coding sequence) is shown for intact genes. Positions of genes carrying inactivating mutations (labeled with "m") indicate the region identified by tBLASTn search using orthologous proteins of other species as queries.

**, RNA-seq peaks in the "Genomic regions, transcripts and products" view at www.ncbi.nlm.nih.gov (accessed on 4 MAY 2021).

***, GenBank gene prediction at the gene locus predicted in the present study

Supplementary Table S6. Cattle (*Bos taurus*) EDC genes that were predicted newly or differently from GenBank

Gene	Accession nr.	CDS start	CDS end	RNA-seq reads*	GenBank Gene ID**	GenBank prediction
LOR	NC_037330.1	17213356	17212343	yes	505154	Different sequence
PRD-SPRRII1	NC_037330.1	17346543	17346752	no		
PRD-SPRRII2	NC_037330.1	17361836	17362051	yes		
PRD-SPRRII3	NC_037330.1	17366591	17366364	yes	100848030	lncRNA
PRD-SPRRII4	NC_037330.1	17389719	17389946	yes	100848441	lncRNA
PRD-SPRRII5	NC_037330.1	17411413	17411640	yes		
PRD-SPRRII6	NC_037330.1	17423061	17423273	yes	112445861	lncRNA
PRD-SPRRII7	NC_037330.1	17432473	17432700	yes	112445862	lncRNA
PRD-SPRRII8	NC_037330.1	17439116	17438895	yes	112445863	lncRNA
PRD-SPRRII9	NC_037330.1	17453412	17453191	yes		
PRD-SPRRII10	NC_037330.1	17457845	17458060	yes		
PRD-SPRRII11	NC_037330.1	17469615	17469836	yes	112445864	lncRNA
PRD-SPRRII12	NC_037330.1	17474433	17474194	no		
PRD-SPRRII13	NC_037330.1	17493330	17493109	yes		
PRD-SPRRII14	NC_037330.1	17530714	17530935	yes	100848091	lncRNA
SPRR2_1	NC_037330.1	17568325	17568513	yes	100848785	lncRNA
PRD-SPRRII15	NC_037330.1	17635776	17635997	yes	112445865	lncRNA
PRD-SPRRII16	NC_037330.1	17658268	17658477	yes		
SPRR2_3	NC_037330.1	17662898	17662707	yes	100848825	lncRNA
SPRR2_4	NC_037330.1	17679315	17679133	yes	107131253	lncRNA
SPRR2_5	NC_037330.1	17699216	17698998	yes	100848624	lncRNA
SPRR2_6	NC_037330.1	17710463	17710218	yes	100848634	lncRNA
SPPR5	NC_037330.1	17805432	17805091	yes		
LCE6A	NC_037330.1	17905065	17904820	yes		
LCE1_1	NC_037330.1	17917346	17917059	yes	104971493	lncRNA
LCE1_2	NC_037330.1	17924326	17924015	yes		
LCE1_3	NC_037330.1	17931004	17930693	yes	786307	Different sequence
LCE1_7	NC_037330.1	17999016	17998705	no		
LCE1_8	NC_037330.1	18007361	18007050	yes	104971494	lncRNA
LCE5A	NC_037330.1	18189257	18188937	yes		

Note: Only gene predictions different from GenBank genes are listed in this table.
Other EDC of cattles were found in GenBank with annotations based on assembly accession GCF_002263795.1
*, RNA-seq peaks in "Genomic regions, transcripts and products" view at www.ncbi.nlm.nih.gov (accessed on 14 July 2020)
**, GenBank gene prediction at the locus of gene predicted in the present study
CDS, coding sequence; lncRNA, long non-coding RNA

A

>Tt_CRCT1
MSSQQTGKDFSKGSSQDTKGPCPTPAPDTSSCCGSGCCRDSGCCGCSSVGCCCLPLRRRLLGSRCCCCCCTDSSQRSRSSSSD
CCGGC

>Tt_IVL (XP_019775380.2)
MSQQCTLPVTLPPAPSKEPLKPVSPPTNIQQEQVKQPTPLPALFQEVSSEIPGKDPMELGKKHTTPVKEVSKKECEPRQQEPQ
QQEQQQQQQKSEEQGKHVEQQQQQKESKEQGKPVEQQQQQKVSQEEGKPVEQQQQQQQQETQEQGKPVEQQQQQKESKEQGKP
VEQQQQQQKESQEQGKPVEQQQQQKESQEQGKPVEQQQQQKESQEQGKPVEQQQQQKESQEQGKPVIQQQQQKVSQEKGKPVE
QQQQKESQEQGKPVEQQQQQKESKEQGKPVEQQQQQQKESQEKGKPVIQQQQQKESQEQGKPVEQQQQKESQEKGKPVEQQQQ
PQKESQEKGKPVEQQQQQQKESQEKGKPVIQQQQQKESQEQGKPVEQQQQKESQEQGKPVEQQQQQQKESQEKGKPVEQQQQQ
QQKESQEKGKPVEQQQQQQQQKESQEQGKPVEQQQQQKVSQEQGKHVEQLKQEKKVLGQQLDQELAKTDEQLEKKGEQQLKQ

>Tt_IVL2
MSQQCTLPVTLPPAPSKEPLKPVSPPTNIQQEQVKQPTPLPAPSQKVPSEIPGKDPMEFGKKHTTPVKEVPKKECEPRQQEPQ
QQEQQQQQQQQQQQQQQKSEEQGKHVEQQQQQQESQEQGKPVEQQQQKKVSQEQGKHVEQQQQQKESQEQGKPVEQQQQQQQS
HRRKESL

>Tt_LCE7A
MSCQQNQQKYQLPAKCLPKYPHKHPPKALQALAPCLPPAPSCCVPSCCISGFGSCCSLVSHRFPSVYLCQPQPSNCCEVEPSG
CSSCCHSFGATADLQPWRSKRATAKEQSGADITSHSRFYSSLSS

>Tt_LOR (XP_019775647.1)
MSCQTKQPTPQPPVGSGKTSGGGGKSGDGGSGGSDGGSGKGVKSSGSCGSSGGGDHSAGGGSSCGGGSSGSDGSGGCRGGSGG
KNSSGGGSSDQKVQGQSHGGVSSGASSGDGSSGCGSGGSQGVPVCHQTQQKQTPSWPCK

>Tt_PRR9 (XP_004331701.1)
MSFNKQQCKQPCMPSPSLQKTQGHCQAKAEEVCLPPCQHSCQKKYPVQAQEVHLPQCQELNQENFLQQGQDPCLPLCQDQSPP
QCVETCQEISQTKRVEVCPQKVQEKCLPPGKGK

>Tt_SPRR2_1
MSSQEQQCKQQCKIPPVCPTKCPDPCSPKKGTDPCQPIKCTEKCTPVPPPEQCQQKCPPKNK

>Tt_SPRR2_2
MSSQEQQCKQQCKIPPVCPTKCPDPCSPKKGTDPCQPIKCTEKCTPVPPPEQCQQKCPPKNK

>Tt_SPRR2_3
MSSQEQQCKQQCKIPPVCPTKCPDPCSPKKGTDPCQPIKCTEKCTPVPPPEQCQQKCPPKNK

>Tt_SPRR2_4
MSSQEQQCKQQCKIPPVCPTKCPDPCSPKKGTDPCQPIKCTEKCTPVPPPEQCQQKCPPKNK

>Tt_SPRR2_5
MSSQEQQCKQRCKLPPVCPAKCPEPCSPTKCTDPCPPIKCTEKCTPVLPPQQCQQKCPPKNK

>Tt_SPRR5_1
MYHPKQKLCCPLPKDCCPPSQQCCTPPKYCCPSPKDCCPLPQQYGPLLRQCCPPVKYCFPPPKHC

>Tt_SPRR5_2
MYHPKQKLCCPLPKDCCPPSQQCCTPPKYCCPSPKYCCPPPQQYCPPPRQCCPPVKYCFPPPKHC

>Tt_SPRR5_3
MYHPKQKLCCPLPKDCCPPSQQCCTPPKYCCPSPKGCCPPPQQYCPPPRQCCPPVKYCFPPPKHC

>Tt_SPRR5_4
MYHPKQKLCCPLPKDCCPPSQQCCTPPKYCCPSPKDCCPPPQQYCPPPRQCCPPVKYCFPPPKHC

>Tt_SPRR5_5
MYHPKQKLCCPLPKDCCPPSQQCCTPPKYCCPSPKDCCPPPQQYCPPPRQCCPPVKYCFPPPKHC

>Tt_SPRRc1
MSSQHQQCKQPCQPPPVCTPKCHDPCPPCKCPDPCPPCKCPDPCPPIKCPDPCVPQQCKQKCPPGAPGQHC

>Tt_SPRRc2
MSSQHQQCKQPCQPPPVCTPKCHDPCPPCKCPDPCPPCKCPDPCPPIKCPDPCVPQQCKQPCQPPPVCTPKCHDSCPPCKYPD
PCSPIKCPDPCVPQQCKQKCPPGAPAHHC

>Tt_SPRRc3
MSSQHQQCKQPCQPPPVCTPKCHDPCPPCKCPDPCPPCKCPDPCPPIKCPDPCVPQQCKQKCPPGAPVQHC

>Tt_SPRRc4
MSSQHQQCKQPCQPPPVCTPKCHDPCPPCKCPDPCPPCKCPDPCPPIKCPDPCVPQQCKQPCQPPPVCTPKCHDPCPPCKCPD
PCPPCKCPDPCPPIKCPDPCVPQQCKQKCPPGAPVQHC

>Tt_SPRRc5
MSSQHQQCKQPCQPPPVCTPKCHDPCPPCKCPDPCPPCKCPGPIPPIKCPDPCVPQQCKQKCPPGAPVQHC

>Tt_SPRRc6
MSSQHQQCKQPCQPPPVCTPKCHDPCPPCKCPDPCPPCKCPDPCPPIKCPDPCVPQQCKQKCPPGAPVQHC

>Tt_SPRRc7
MSSQHQQCKQPCQPPPVCTPKCHDPCPPCKCPDPCPPCKCPDPCPPIKCPDPCVPQQCKQKCPPGAPVQHC

B

>Tt_FLG
MSTLLENITAIIKLFHEYSKTDKETDTLSAKELKELLEAEFQPILKNPDDPDTADVFMHILNVDHNHKIDFTEFFLMVFKLAQ
AYYYTQRPNFKTLGKKQKKNRYHYEDDTEEEGKEERERKSSHSRSDGKKEDRTEEEEEGRSRHGSSSGREGRHRNRSGKKRHE
SSREKKRRASSTELKERSHMSSVSPIREYEGKEEERGYENKDRGCEKWIGSEPKGSYQVCEETVTMDFQSGCSTQQVSSISKG
SDSKEHSQDSGRQPVITHGRSPSSSRNQHGSSHDQSGDSPKHSESHQGRTDTHRKSESVHGKSGSSTTQRQGRHHEQEKDSSR
PSGTGHGHASTGSGSSRHRESSVGQSSDSEIQSGNSGRYSVTTHGRSGSSSKNQHGSSQGQSRDSSRHSESHQGRTDTHKKSE
SGHSKSGSSTTQRQGHHHEQERDSSRHSGTGHGHTSDGSRSGKDRESSVGQSSDSEIQSGNSGRYSVTTHGRSGSSSKNQHGS
SQGQSRDSSRHSESHQGRTDTHKKSESGHSKSGSSTTQRQGHHHEQERDSSRHSGTGHGHTSDGSRSGKDRESSVGQSSDSEG
QSEDSHRHSVTTHGRSGSSSRNQHGSSHGRSGDSSRHSESHQGRTDTHRKSESVHGESGSSTKQRQGHHHEQEKDNSRHSGIG
HGHASTGSRKSKQRESSVGQSSDSEGQSEDSGRHSVTTHGRSGSRSRNQHGSSHGWSGDSSRHSESHQGRSAYRKSESGHSKS
GSSTTQRQGHHHEQERDSSRYSGTGHGHTSTGSGSNRHRESSVGWSSDSEGQSGDSGRHSVTTHGGSGSSSRNQHGSSHGWSG
DSSRHSESHQGRSAYRKSESGHSKSGSSTTQRQGHHHEQERDSSRYSGTGHGHTSTGSGSNRHRESSVGWSSDGEGQSGDSSR
HSVTTHGRSGSSSRNQHGSSHGQSGDSSRHSESHQGRTDTHKKSESGHSKSGSSTTQRQGHHHEQERDSSRHSGTRHGHTSTI
SGSGRHKESNISQASNTEGHSGDSGRQPLTTQGWSAFYSRNQSHGSDQGWRHGSYGSADYDYGQSGFGHSQDGSVSHDSSHMG
ARDRFEYRSIYGIQYNRQ

C

>Tt_PGLYRP3
MLLWLLLISALDLGAWGDYPQLSWNETQARGLSEKLLDLFVGISQLILKGRSGASTIVSRKEWGLRFLTRRAQLTRPVACVIM
DQLTEVECQEQNVCSWKLRGLQSRSVYNTGWCDVAYIFLVGDNGRVYKGVGWNIQGMHAQGYNSVSLGLAFFGNKLGSRPSPA
ALSAAEDLIFYAIKKGHMSLRYIQPLLLKEESCLVPQQPLMPRKACPNIITRSAWKARQTHCPTMKLPAKYVIIIHTVGATCN
ISMDCRICV

>Tt_S100A9 (XP_004329971.1)
MADQLSQLESSIETIINIFHQYSIRLQPPDTLNKKEFKQLVKKELPNFLKKETKDDKAINEIMEDLDTDVDKELNFHEFSVLV
GKLTEASHEEMHKTAPPGVGHRHGPGFGAGGSGHGHSHDNHGHSHGNHGHSH

>Tt_S100A11 (XP_004324172.1)
MAKTSSPTETERCIESLIAVFQRHAGRDGNNSKLSKAEFLIFMNTELGAFTKNQKDPGVLDRMMKKLDLDSDGQLDFQEFLNL
IGGLALACHDSFIKSTSSRK

Supplementary Figure S1. Amino acid sequences of proteins encoded by EDC genes of the bottlenose dolphin. (A) Amino acid sequences of proteins encoded by SEDC genes of the dolphin. **(B)**

Amino acid sequences of dolphin SFTPs. **(C)** Amino acid sequences of proteins encoded by other EDC genes of the dolphin. To show the peculiar amino acid compositions of SEDCs and SFTPs and the importance for protein cross-linking the following amino acid residues are highlighted: lysine (K) and glutamine (Q) as potential sites of transglutamination; cysteine residues (C) as potential sites of disulfide bonds; glycine (G), proline (P) and serine (S) are highly abundant residues not directly involved in cross-linking. When available, the GenBank accession number is shown behind the protein name. “XXX” indicates a stretch of unknown amino acid residues, corresponding to a gap in the gene sequence. Only the S100A proteins whose genes are flanking *PGLYRP3* and *FLG* are included here. SEDC, Simple epidermal differentiation complex gene; SPRR, small proline rich protein; SFTP, S100 fused-type protein; Tt, *Tursiops truncatus*.

A

>Ps_CRCT1
MSSQQSGKDFSKGSSQDTKGPCPTPAPDTSSCCGSGCCGDSGCCGSSSVGCCCLPLRRRLLGSRCCCCCADSSQRSRSSSSDC
CGGC

>Ps_IVL
MSQQRTLPVTLPPAPSKESLKPVSPPTNIQQEQAKQPTPLPAPFQKVSSEFPGKDPMELGKKHTTPVKEVPKKECEPRQQEPQ
QQQQQQQQQQESQKPGKPVDQQQQQKESQEPGKPVDQQQQQKESQEPGKPVDQQQQQKESQEPGKPVDQQQQQKESQKPGKHV
QQQQQQKESQEPGKHVEQQQQQKESQEPGRPVDQQQQQKESQKPGKPVDQQQQQKESQKPGKPVDQQQQQQKESQEPGKPVDQ
QQQQKESQEPGKPVDQQQQQKESQEPGKHVQQQQQQKESQEPGKHVEQQQQQKESQEPGKPVDQQQQQKESQEPGEPVDQQQQ
QKESQEPGKPVDQQQQQKESQEPGKPVDQQQQQKESQEPGKPVDQQQQQKESQEPGKHVQQQQQQKESQQPGKPVDQQQQQKE
SQEPGKHVQQLKQEKKVLGQRLDQELAKKDQQLEKKGEQQLKQQNGLLK

>Ps_IVL2
MSQQLTLPVTLPPAPSKESLKPVSPPTNIQQEQAKQPTPLPAPFQKVSSEFPGKDPMEVGKKHTTPVKEVPKKECEPRQQEPQ
QQQQQQQQQQESQKPGKPVDQQQQQKESQEPGKPVDQQQQQKESQEPGKPVDQQQQQKESQEPGKPVDQQQQQKESQKPGKHV
QQQQQQKESQEPGKHVEQQQQQKESQEPGKPVDQQQQQKESQKPGKPVDQQQQQKESQKPGKPVDQQQQQKESQEPGKPVDQQ
QQQKESQEPGKPVDQQQQQQKESQEPGKPVDQQQQQKESQEPGKPVDQQQQQKESQEPGKPVDQQQQQKESQEPGKPVDQQQQ
QKESQEPGKHVDQQQQQKESQEPGKHVQQLKQEKKVLGQRLDQELA

>Ps_LCE7A
MSCQQNQQKCQLPAKCLPKYPPKHPPKAPQALAPCPPPSPSCSVPSCCISGFGSCCSLVSHRFPSVHLCQPQPSNCCEVELSG
CSSCCHSFGATADLQPWLEQKSHSQRTVWS

>Ps_LOR
MSHQTKQPTPHPPVGSGKTSGGGGKSGDGGCGGSDDGSGKSVKSSGSCGSFSGGDHSAGGGSSCGGGSSGSDGSGGCRGGSGG
KNSSGGGSSDQKGQGQSYGGVSSGGSCGDGSSGGGSGGSQGVPVCHQTQQKQTPTWPSK

>Ps_SPRR2_1
MTYKQHQCKQPCQSPLVFTPKCPEPCPPKCPEPYPHKCSEPCPPLNCLEPCPCPPCPPVQCHIHCASRNVLLCIHAHPASRSC
PPKYK

>Ps_SPRR2_2
MSSQEQQCKQQCKIPPVCPTQCPDPCSPKKGTDPCPPIKCTEKCTPVPPPQQCQQKCPPKDK

>Ps_SPRR2_3
MSSQEKQCKQQCKIPPVCPTKCPDPCSPIKGTDPCPPIKCTEKCTPVPPPQQCQQKCPPKDK

>Ps_SPRR2_4
MSSQEKQCKQQCKIPPVCPTKCPDPCSPIKGTDPCPPIKCTEKCTPVPPPQQCQQKCPPKDK

>Ps_SPRR2_5
MSSQEKQCKQQCKLPPVCPTKCPDPCSPKKGTDPCPPIKCTEKCTPVPPPQQCQQKCPPKDK

>Ps_SPRR2_6
MSSQQQQCKQPCQPPPVCPPKCPESCPPKCSDLCPPIKCPEPCLPQHCQQKCPSVPLLQQCQQKCPPNNK

>Ps_SPRR3
MSSYQQKKPCIPPHEPQQQEVKQPCQPPPQEPCVPKNKEPCHTKMPEPCHPKIPEPCQPKFPEPCHPKVPEPCHTKVPEPCHP
KAPEPGHPKVPEPCPSPVIPGPAQQKTKQK

>Ps_SPRR5_1
MYHLEQKLCCPLPKYCCPPPQPCWTPPKYCCPSPKYCCPPPQQYCPPPRQCCPPVKYCFSPPKHC

>Ps_SPRR5_2
MYHPKQKLCCPLPKYFCPPPQPCWTPPKYCCPSLKYCCPPPQQYCPSPQLWWSQAKYCFPPPKHC

>Ps_SPRR5_3
MYHLEQKLCCPLPKYCCPPPQPCWTPPKYCCPSPKYCCPPPQQYCPPPQQCWPRAQYCFPPPKHC

>Ps_SPRR5_4
MYHLKQKLCCPLPKYCCPPPQPCWISPNYCCPSPKDCCPPPQQYYSGPQLWWSRAQYCFSPPKHC

>Ps_SPRR5_5
MYHPKQKLCCPLPKYCCPSPKYCCPPPQQYCPPPRQCCPPVKYCFPPPKHC

>Ps_SPRR5_6
MYHPKQKLCCPLPKCCCPPTQPCWTPSNYCCLSLKYCCPPPQQYCPPPRQCCPPVKYCFSPPKHC

>Ps_SPRR5_7
MYHPKQKLCCPLPKCCCPPPQPCCTPSNYCCPSPKYCCPPPQQYCPPPRQCCPPVKYCFSPPKHC

>Ps_SPRR5_8
MYHPKQKLCCPLPKCCCPPPQPCCTPSNYCCPSPKYCCPPPQQYCPPPRQCCPPVKYCFSPPKHC

>Ps_SPRRc1
MSSQHQQCKQPCQPPLVCTPKCHDPCPACKCPDPCSPCKCPDPCPPIKCPDPCVPLQCKQKCPPVARAHHC

>Ps_SPRRc2
MSSQHQQCKQPCQPPPGCTPKCHDPCSPWKGPDPCSPCKCPDPCPPIKCPDPCLLQQCKQKCPPGSPAHHC

>Ps_SPRRc3
MSSQHQQCKQPCQPPPVCTPKCHDPCPPCKCPDPCPPCKCPDPCPPIKCPDPCVPQQCKQKCPPGARAHQR

>Ps_SPRRc4
MSSHHQQCKQPCQPPPVCTPKCPDPCPPCKCPDPCPPCKCPDPCRSVKCPDPCVPQQCKQKCPPGAPAHHC

>Ps_SPRRc5
MSSQHQQCKQPCQPPPVCTPKCHDPCPPCKCPDPCPPCKCPDPCPPIKCPDPCVPQQCKQKCPPGARAHQR

>Ps_SPRRc6_partial
MSSQHQQCKQPCQPPPVCTPKCHDPCPPCKCPDPCPPCKCPXXX

>Ps_SPRRc7
MSSQHQQCKQPCQPPPVCTPKCHDPCPPCKCPDPCPPCKCPDPCPPIKCPDPCVPQQCKQKCPPGAPVHQC

B

>Ps_FLG
MSTLLENITAIIKLFHEYSKTDKETDTLSAKELKELLEAEFQPILKNPDDPDTADVFMHILDVDHNNKIDFTEFFLMVFKLAQ
AYYYTQRPNFKTLGKKQKKNRYHYEDDTEEEDKEERERKSSHSRRSDGKKKDRTESPRGRGRSRHGSSSGREGRRGDRATSGH
RNRSGKKHHESSREKKRRASPTELKERSHMSSVSPIREYEGKEEERGYENKGRECEKWIGSEPKGSYQVCEETVTMDFQSGCS
THQVSSISKGSDSKEHSQDSGRQPVITHGRSPSSSRNQHGSSHDQSGDSPKHSESHQGRTDTHRKSESVHGKSGSSTTQRQGR
HHEQEKDSSRHSGTGHGHASTGSGSNRHRESSVGQSSDSEIQSGDSGRYSVTTHGRSGSSSKNQHGSSHGQSGDSSRHSELHQ
GRTDTHRKSESVHGKSGSSTTQRQGLHHEQERDSSRYSGTGHGHTSDGSRSGKDRESSVGQSSDSEGQSEDSDRHSVTTHGRC
GSSSRNQHGSSHGWSGDSSRHSESHQGRSAYRMSESGHSKSGSSTTQRQGHHHEQERDSSRYSGTGHGHTSTGSGSNRHRESS
VGWSSDGEGQSGDSSRHSVTTHGRSGSSSRKQHGSSHGQSGDSSRHSESHQGRTDTHKKSESGHSKSGSSTTQRQGHHHEQES
DSSRHSGTGHGHTSTISGSGRHKESNISQASNTEGHSGDSGRQPLTTQGWSAFYSRNQSHGSDQGWRHGSYGSADYDYGQSGF
GHSQDGSVSHDSSHMGARDRFEYRSIYGIQYNRQ

C

>Ps_PGLYRP3
MLLWLLLISALDLGAWGDSPQLSWNETQARGLSEKLLDLFVGISQLILKGRSGASTVVSHKEWGLRFLTCRAQLTRPVACVIM
DQLTEIECQEQNVCSWKLRGLQSRSVYNTGWCDVAYIFLVGDNGRVYKGVGWNIHGMHAQGYNSVSLGLAFFGNKLGSRPSPA
SLSATEDLIFYAIKKGHVSLRYIQPLLLKEESCLVPQQPLMPRKACPNIITRSAWKARQTHCPTMNLPAKYVIIIHTIGATCN
ISMDCRICV

>Ps_S100A9 (XP_032460313.1)
MADQLSQLESSIETIINIFHQYSIRLQPPDTLNKKEFKELVKKELPNFLKKETKDDKAINEIMEDLDTDVNKELNFQEFSVLV
GKLTEASHEEMHKTAPPGVGHRHGPGFREGGSGHGHSHGNHGHSH

>Ps_S100A11 (XP_032467955.1)
MAKTSSPTETERCIESLIAVFQRHAGRDGNNSKLSKAEFLIFMNTELGAFTKNQKDPGVLDRMMKKLDLDSDGQLDFQEFLNL
IGGLALACHDSFIKSTSSQK

Supplementary Figure S2. Amino acid sequences of proteins encoded by EDC genes of the vaquita porpoise. **(A)** Amino acid sequences of proteins encoded by SEDC genes of the porpoise. **(B)** Amino acid sequences of porpoise SFTPs. **(C)** Amino acid sequences of proteins encoded by other EDC genes of the porpoise. To show the peculiar amino acid compositions of SEDCs and SFTPs and the importance for protein cross-linking the following amino acid residues are highlighted: lysine

(K) and glutamine (Q) as potential sites of transglutamination; cysteine residues (C) as potential sites of disulfide bonds; glycine (G), proline (P) and serine (S) are highly abundant residues not directly involved in cross-linking. When available, the GenBank accession number is shown behind the protein name. “XXX” indicates a stretch of unknown amino acid residues, corresponding to a gap in the gene sequence. Only the S100A proteins whose genes are flanking *PGLYRP3* and *FLG* are included here. SEDC, Simple epidermal differentiation complex gene; SPRR, small proline rich protein; SFTP, S100 fused-type protein; Ps, *Phocoena sinus*.

A

>Ba_CRCT1
MTSQQSGKDFSKGSSQDAKSSCPTPVPDASSCCGLGCCGDSGCCGVSSAACCCFPVRRRLQGSRGCCCCCADSSQSSGSSSSG
CCGAC

>Ba_IVL
MSEQGTLPVTLPPALCKEPVKPVSPPTNIQQEQVKQPTPLPAPCQKVIPGKDPLELGEKHTTPVKEVPEQGCKPQQQEPEHPE
QHPEQQQQQQQQESQKQEKHVEQQQQQQQQESQKQEKHVEQQQQQQQQESQKQEKHVEQQQQQQQQESQKQEKHVEQQQQQQQ
QESQKQEKHVEQQQQQQQQESQKQEKHVEQQQQQQQQESQKQEKHVEQQQQQQQQESQKQEKHVEQQQQQQQQESQKQEKHVE
QQQQQQQQESQKQEKHVEQQQQQQQQESQKQEKHVEQQQQQQQQESQKQEKHVEQQQQQQQQESQKQEKHVEQQQQQQQQESQ
KQEKHVEQQQQQQQQESQKQEKHVEQQQQQQQQESQKQEKHVEQQQQQQQQESQKQEKHVEQQQQQQQQESQKQEKHVEQQQQ
QQQQESQKQEKHVEQQQQQQQQESQKQEKHVEQQQQQQQQESQKQEKHVEQQQQQQQQESQKQEKHVEQQQQQQQQESQKQEK
HVEQQQKQQESQKQEKHVEQQQQQQESQKQDQHVQQLEQEKKVLGQRLDQEPAKKDEQLEKKGEQLLVQQEGPLKQPLLVPAP
GQV

>Ba_LELP1 (XP_007178639.1)
MSSDDKNKPSEPKNEPKQCDPRCEQRCETKCQPSCLKKLLQRCSEKCPREKCPAPPKCPLCPPCPPLCPPPCPRPAPSPPKPC
SKPCPPKCPPPCPPPE

>Ba_LOR_partial
MSHQTKQPTPQPPVGSGKTSGGGGSSGGGGSSGGGGSSGGGGSGGXXXGGGSGGSDGGSGGKVKHSGSGGSSGGGSSGGGGSS
GSDGSGGCGGGTSGKNSSGGGSSGGGSGDGQGFPVCHQTQQKQTPTWPSK

>Ba_SMCP
MCDQPKCNPSCPPKCNTCCPPKPPCYIQPTCCCLEPKPECTCLNKETDPTAPQTQNNNSLSQQQSQSPKLAPKSGPPGAEAAE
QVDCHKQVDSAMPAAW

>Ba_SPRR2_1 (XP_007178639.1)
MSSEQQQCTQPCQPPPVCPPKGPDPCSPPKCPEPCPRIKCPEPCPPQQCQQKCPPVPPPQQCQQKCPPKNK

>Ba_SPRR2_2
MSSQEQQCKQKCKTTPVCVTKCPDPCSPKKDSDPCPPQPCQQKYPPGTPIQQCQQKCPPKEK

>Ba_SPRR2_3
MSSQEQQGKQKCKSPLVCVTKCPDPCSPKKDSDPCPPQPCQQKCPPGTPIQQCQQKCPLKSK

>Ba_SPRR2_4 (XP_028019918.1)
MSSQEQQCKQKCKTPPVCVTKCPDPCSPKKDSDPCPPQPCQQKCPPVTPTQKCQQKCPPKDK

>Ba_SPRR2_5 (XP_028024403.1)
MSSQEQQCKQKCKTPPVCVTKCPDPCSPKKDSDPCPPQPCQQKCPPVTPTQQCQQKCPPKNK

>Ba_SPRR2_6 (XP_007190509.1)
MSSQEQQCMQQCKTPPVCATKCPDPCSPPKCPDPCPPIKCPEPCPPQQCQQKRPTVPPPKQCQQKFPPKNK

>Ba_SPRR2_7
MSSQHQQCKQPCQPPPVCPPKCPEPCPPKCPELCPPIRCPEPRPPQQCQQKCPPVPLPQLPTEVPTQ

>Ba_SPRR3 (XP_028024313.1)
MSSYQQKQPCIPPHEPQQQQVKQPCQPPPQEPCVPETKETCHTKVPEPCHPKVPEPCQPKVPEPGHPKVPEPGHPKVPEPGHP
KVPEPSHPKVPEPCPSPVIPGPAQQKTKQK

>Ba_SPRR4 (XP_028024312.1)
MSSWQQQQQQEHQCPPQVAQRQQVKQLCQPPPVKCQETCVPKTKDPYAPQAKKQCPPKGTPIPAQQKCPSAQQAPKSK

>Ba_SPRR5_1
MYHQKQKLCCPASQPCCPPPKYCCPSPQPCCPLPKYCCPGPQQYCLPTQCCCLPPKQC

>Ba_SPRR5_2_partial

MYHQKQKLCFPPPESSFPAPQQRCPLPKYCCPTPQHSCPLPKYCCXXX

>Ba_SPRR5_3
MYHPKQKLCCPLPKYCCPPSQQCCPPPKYCCPAPQPCCPLPKYCCPPLQQYCPPLQQCCPPAGYRFPSPKQF

>Ba_SPRR5_4
MYHQKQKLCFRPPKSCFPAPQPCCPLPKYCCPAPQQSCPLPKYCCPAPQQSCPLPKYCCPAPQQSCPLPKYCCPAPQQSCPLP
KYCCPAPQQYCPPPQ

>Ba_SPRRc1
MSSQHQQCKQPCQPPPVCLPKCPDPCPPAKCPDPCPPIKCPDPCPPAKCPDPCAPIKCPDPCLPQQCKQKCPPGPPAHHC

>Ba_SPRRc2
MSSQHQQCKQPCQPPPVCLPKCPDPCPPAKCPDPCPPIKCPDPCPPAKCPDPCAPIKCPDPCLPQQCKQKCPPGPPAHHC

>Ba_SPRRc3
MSSQHQQCKQPCQPPPVCLPQCPDPCPPAKCPDPCPPIKCPDPCPPQQCKQKCPPGPPAHHC

B

>Ba_S100A9 (XP_028021335.1)
MNDNESPGNPANSGVVPVGPEGHILTSGNVLPMGTDLLQEFEGCECRKITDTMSQLESSVETIINIFHQYSVRLQPPDTLNHK
EFKQLVEKELPNFLKHKDDQAINKIMEDLDTDVDKQMDFEEFLILVAGLTEASHEEMHNTAPKVSGHSHGPGFEKGGSGPCPS
KVTQSPCHGDHSHSHSHGNHGHSH

>Ba_S100A11 (XP_007178142.1)
MAKTSSPTETERCIESLIAVFQKHAGRDGDNCKLSKAEFLIFMNTELGAFTKNQKDPCVLDRMMKKLDLDCDGQLDFQEFLNL
IGGLALACHDSFIKKSTPSHK

Supplementary Figure S3. Amino acid sequences of proteins encoded by EDC genes of the minke whale. (A) Amino acid sequences of proteins encoded by SEDC genes of the whale. **(B)** Amino acid sequences of proteins encoded by other EDC genes of the whale. To show the peculiar amino acid compositions of SEDCs and SFTPs and the importance for protein cross-linking the following amino acid residues are highlighted: lysine (K) and glutamine (Q) as potential sites of transglutamination; cysteine residues (C) as potential sites of disulfide bonds; glycine (G), proline (P) and serine (S) are highly abundant residues not directly involved in cross-linking. When available, the GenBank accession number is shown behind the protein name. "XXX" indicates a stretch of unknown amino acid residues, corresponding to a gap in the gene sequence. Only the S100A proteins whose genes are flanking *PGLYRP3* and *FLG* are included here. SEDC, Simple epidermal differentiation complex gene; SPRR, small proline rich protein; SFTP, S100 fused-type protein; Ba, *Balaenoptera acutorostrata scammoni*.

>Bt_LCE1_1
MSCQQNQQQCQPPPKCTPKCHTPKCPPKCPPVSSCCGPSSGDCCSSGCGSCCLSHHRRRRSHHCRPHRSDCCSQPSGGSGCCG
EGSGQSFGGGCC

>Bt_LCE1_2
MSCQQNQQQCQPPPKCVPKCPTPKCPPKCPQVSSCCDVSSEGCCGPSSGGCCSSGCGGCCLSHHRRRRSHHCRPHRSDCCSQP
SGGSGCCGGGSGQSYGGGCC

>Bt_LCE1_3
MSCQQNQQQCQPPPKCVPKCPTPKCPPKCPPVSSCCGVSSEGCCGPSSGGCCSSGCGGCCLSHHRRRRSHHCRPHRSDCCSQP
SGGSGCCGGGSGQSYGGGCC

>Bt_LCE1_7
MSCQQNQQQCQPPPKCAPKCPTPKCPPKCPPVSSCCDVSSEGCCGSSSGDCCSSGCGGCCLSHHRRRRSHHCRPHRSDCCSQP
SGGSGCCGGGSGQSFGGGCC

>Bt_LCE1_8
MSCQQNQQQCQPPPKCAPKCPTPKCPPKCPPVSSCCDVSSEGCCGSSSGDCCSSGCGGCCLSHHRRRRSHHRRRHRSDCCSQP
SGGSGCCGGGSGQSFGGGCC

>Bt_LCE5A
MSCQQNQQQCQPPPKCPPKCPPKCTPKCPPKCPAPCPPPASSCCAPSSEGCCSSGGGGCCLSHHKLRRSLRRRHQSSGCCGSG
GGQHSRDSGCCSGGSSCCSGGCC

>Bt_LCE6A
MSQQNQQNHGSPGALRCPPPQRPTPFPVSCSAPCPPYAGHCGSGSQGIRPRDQSLAGSWRALWKPRCLSGGTTHHIKEEEC

>Bt_LOR
MSHQKKQPTPQPPVGCGKTSGGSGGVFYSGGGGGSGSCGSSGGGGGGGCSGGSIKYSGGGGSSGCEGYSSGGGSSGTVCYPSG
GGSSGTVCYPSGGGSSGTICHSSGGGSSGTVCYPSGGVSSGTVCHSSGGASSGTVCYPSGGVSSGTVCHSSGGGSSGTVCYSS
GGGGSSGQQVQCQSYGGVSSGGGSACGVISSGDGSGCGGISSGGGCGAISSGGGSGCGGISSGGGSGYFSSSQTSQTPCLPQE
SYGGGSSGAGCGGGSSGVSGCFSSGGGGSGSVCLGGSSGGGGGCISSGGGGSGSGCGGGSSGGASGGGKGVPVCHQTQQKQAP
TWPGK

>Bt_PRD-SPRRII1
MSHHQHPHLHQHQHHHQCKEPCHPPPIVCPPKCHEPCPPHSCPPSLSQQKCPPGPPYPPCKQKCPPKSK

>Bt_PRD-SPRRII2
MSQQQHPHPHQHQHQHHHQCKEPCHPPPKVCPPKCHEPCPPHPCPPPLGQKKCPPGPPCPPCEQKCPPKWK

>Bt_PRD-SPRRII3
MSHHPHPHPHPHPQKHQHQHHHQCKEPCHPPPIVCPPKCHEPCPPHPCPPPVSQQKCPPRPPCPPCEQKCPPKWK

>Bt_PRD-SPRRII4
MSHHPHPHPHPHPHPHPHQHHHQCKVPCHPPPKVCPPKCHEPCPPHPCPSPPSQKKCPPG PPCPPCKQKCPPKWK

>Bt_PRD-SPRRII5
MSHPPHPHPHPHPHPHPHQHHHQCKEPCHPPPKVCPPKCHEPCPPHPCPSPPSQKKCPPGPPCPPCKQKCPPKWK

>Bt_PRD-SPRRII6
MSHHQHPHPHQNQHQHHHQCKEPCHPPPKVCPPKCHEPCPPHPCPPPLSQKKCPPGPPCPPCEHKCPPKWK

>Bt_PRD-SPRRII7
MSHHPHPHPHPHPHPHQHQHHHQCKEPCHPPPKVCPPKCHEPCPPHPCPPPVSQQKCPPRPPCPPCEQKCPPKWK

>Bt_PRD-SPRRII8
MSNHPHPHPHPHQHQHQHHHQCKEPCHPPPKVCPPNCHEPCPPHPCPPALSQKKCPPGPPCPPCEQKCPPKWK

>Bt_PRD-SPRRII9
MSHHQHPHPHPHQHQHQHHHQCKEPCHPPPKVCPPKCHEPCPPHPCPPPLSQKKCPPGPPCPPCEHKCPPKWK

>Bt_PRD-SPRRII10
MSHHPHPHPHPHPHPHPHQCKEPCHPPPKVCPPKCHEPCPPHPCPPPLGQKKCPPGPPCPPCEQKCPPKWK

>Bt_PRD-SPRRII11
MSHHPHPHPHPHQHQHQHHHQCKEPCHPPPIVCPPKCHEPCPPHPCPPPLGQKKCPPGPPCPPCEQKCPPKWK

>Bt_PRD-SPRRII12
MSHHKHPHPHPHQHKHQHHHQCKEPCHPPPIVCPPKCHEPCPPHPCPPPLSQQKCPPGPPCPPCNQKCPPSEQKCPPKWK

>Bt_PRD-SPRRII13
MSNHPHPHPHPHQHQHHHHHQCKEPCHPPPKVCPPKCHEPCPPHPCPSPPSQKKCPPGPPCPPCEQKCPPKWK

>Bt_PRD-SPRRII14
MSHHPHPHPHPHPHPHQHHHQCKEPCHPPPKVCPPKCHKPCPPHPCPPPLGQKKCPPGPPCPPCEQKCPPKWK

>Bt_PRD-SPRRII15
MSYHQHPHPHPHQHQHQHHHQCKEPCHPPPIVCPPKCHEPCPLHPCPPPVSQKKCPPGPPRPPWDQKCPLKWK

>Bt_PRD-SPRRII16
MSHHQHPHQHPHQHHHQCKEPCHPPPIVCQPKCHEPCPPHPCPPPLSQQKCPPGPPCPPWEQKCPPKWK

>Bt_SPRR2_1
MSQQQQQCKQPCQPPPVVCPPKCPEPCPPKCPEPCPPPKCQEKCPPVPPPQQCQQKCPPKCK

>Bt_SPRR2_3
MSQQQQQQQQQQQQQQCKQPCQPPPVVCPPKCPEPCPPPKCQQKCPPVPPPQQCQEKCPPKCK

>Bt_SPRR2_4
MSQQQQQQQQQQQQCKQPCQPPPVVCPPKCPEPCPPPKCQQKCPPVPPPQQCQEKCPPKCK

>Bt_SPRR2_5
MSYQQQQCKQPCQPPPVVCPPKCPEPCPPPKCPEPCPPPKCPEPCPPPKCQQKCPPVPPPQQCQEKCPPKCK

>Bt_SPRR2_6
MSYQQQQCKQPCQPPPVVCPPKCPEPCPPPKCPEPCPPPKCPEPCPPPKCPEPCPPPKCQQKCPPVPPPQQCQQKCPPKYK

>Bt_SPRR5
MSQQKQKQCAPPQLCCPPVKQCCPPPQQCCPPPQQYCPPPQQCCPPPKQCCPPPQKCCPPPQQCCPPPQKCCPPPQKCCPPPQ
QCCPPPKQCCPPPKQCCPPPKHCCPPPKQC

Supplementary Figure S4. Amino acid sequences of proteins encoded by EDC genes of cattle. Amino acid sequences of proteins encoded by cattle SEDC genes that were predicted in this study. GenBank annotations were used for all other cattle EDC proteins which are not shown here. To indicate the peculiar amino acid compositions of SEDCs and SFTPs and the importance for protein cross-linking the following amino acid residues are highlighted: lysine (K) and glutamine (Q) are potential sites of transglutamination; cysteine residues (C) are potential sites of disulfide bonds; glycine (G), proline (P) and serine (S) are residues highly abundant in some SEDC proteins. SPRR, small proline rich protein, LCE, late cornified envelope. Bt, *Bos taurus*.

```
>Cattle_IVL
MSQQHTLPVIQPPALSQESLKPASPPTNTQQ
EQVKQPTPLPAPCQKVHSELPGEVPLELGEK
HTIVKGVAEQKCEPQQP
EPEQQEQHVEQQQQ
ESQVQEQNVEQHQQ
ESQVQEQYVEEQQQ
ESQVQEVHVEQQQQ
ESQVQEQHVEQQQQ
ESQVQEQHVEQQQQ
ESQVQEQHVEQQQQ
ESQVQEQHVEQQQQ
ESQVQEQHVEQQQQ
ESQVQEQHVEQQQE
ESQVQEQYVEQQQQ
ESKVQEQHVKQQQQ
ESQVPEEHVKQQQQ
ESQVPEVHVEQQQQ
ESQVPEKHVELQQQ
ESQVPEVHVELQQQ
ESQVPKVHVKKQHQ
ELQEQQVQQQKEQHVEHQEAEHLGLQLEQEK
TQREQQLKGQLEQEKKVLGQHLDQEPAKREE
QMEKKEEQLLEQQEGQLKQPVFVPAPGQVQE
THPVLPLKEKALPSEEAGGVVPSKHK
```
repeats

```
>Bottlenose_dolphin_IVL1
MSQQCTLPVTLPPAPSKEPLKPVSPPTNIQQ
EQVKQPTPLPALFQEVSSEIPGKDPMELGKK
HTTPVKEVSKKECEP
RQQEP-QQQEQQQQQQ--K
-SEEQGKHVEQQQQQ---K
ESKEQGKPVEQQQQQ---K
VSQEEGKPVEQQQQQQQQ-
ETQEQGKPVEQQQQQ---K
ESKEQGKPVEQQQQQQ--K
ESQEQGKPVEQQQQQ---K
ESQEQGKPVEQQQQQ---K
ESQEQGKPVEQQQQQ---K
ESQEQGKPVIQQQQQ---K
VSQEKGKPVEQQQQ----K
ESQEQGKPVEQQQQQ---K
ESKEQGKPVEQQQQQQ--K
ESQEKGKPVIQQQQQ---K
ESQEQGKPVEQQQQ----K
ESQEKGKPVEQQQQPQ--K
ESQEKGKPVEQQQQQQ--K
ESQEKGKPVIQQQQQ---K
ESQEQGKPVEQQQQ----K
ESQEQGKPVEQQQQQQ--K
ESQEKGKPVEQQQQQQQ-K
ESQEKGKPVEQQQQQQQQK
ESQEQGKPVEQQQQQ---K
VSQEQGKHVEQLKQE---K
KVLGQQLDQELAKTDEQLEKKGEQQLKQ
```
repeats

```
>Vaquita_IVL1
MSQQRTLPVTLPPAPSKESLKPVSPPTNIQQ
EQAKQPTPLPAPFQKVSSEFPGKDPMELGKK
HTTPVKEVPKKECEP
RQQEPQQQQQQQQQQ-
ESQKPGKPVDQQQQQ-K
ESQEPGKPVDQQQQQ-K
ESQEPGKPVDQQQQQ-K
ESQEPGKPVDQQQQQ-K
ESQKPGKHVQQQQQQ-K
ESQEPGKHVEQQQQQ-K
ESQEPGRPVDQQQQQ-K
ESQKPGKPVDQQQQQ-K
ESQKPGKPVDQQQQQQK
ESQEPGKPVDQQQQQ-K
ESQEPGKPVDQQQQQ-K
ESQEPGKHVQQQQQQ-K
ESQEPGKHVEQQQQQ-K
ESQEPGKPVDQQQQQ-K
ESQEPGEPVDQQQQQ-K
ESQEPGKPVDQQQQQ-K
ESQEPGKPVDQQQQQ-K
ESQEPGKPVDQQQQQ-K
ESQEPGKHVQQQQQQ-K
ESQQPGKPVDQQQQQ-K
ESQEPGKHVQQLKQE-K
KVLGQRLDQELAKKDQQLEKKGEQQLKQQNG
LLK
```
repeats

```
>Minke_whale_IVL
MSEQGTLPVTLPPALCKEPVKPVSPPTNIQQ
EQVKQPTPLPAPCQKVIPGKDPLELGEK
HTTPVKEVPEQGCKPQQQ
EPEHPEQHPEQQQQQQQQ
ESQKQEKHVEQQQQQQQQ
ESQKQEKHVEQQQQQQQQ
ESQKQEKHVEQQQQQQQQ
ESQKQEKHVEQQQQQQQQ
ESQKQEKHVEQQQQQQQQ
ESQKQEKHVEQQQQQQQQ
ESQKQEKHVEQQQQQQQQ
ESQKQEKHVEQQQQQQQQ
ESQKQEKHVEQQQQQQQQ
ESQKQEKHVEQQQQQQQQ
ESQKQEKHVEQQQQQQQQ
ESQKQEKHVEQQQQQQQQ
ESQKQEKHVEQQQQQQQQ
ESQKQEKHVEQQQQQQQQ
ESQKQEKHVEQQQQQQQQ
ESQKQEKHVEQQQQQQQQ
ESQKQEKHVEQQQQQQQQ
ESQKQEKHVEQQQQQQQQ
ESQKQEKHVEQQQQQQQQ
ESQKQEKHVEQQQQQQQQ
ESQKQEKHVEQQQQQQQQ
ESQKQEKHVEQQQQQQQQ
ESQKQEKHVEQQQKQQ--
ESQKQEKHVEQQQQQQ--
ESQKQDQHVQQLEQEKKVLGQRLDQEPAKKD
EQLEKKGEQLLVQQEGPLKQPLLVPAPGQV
```
repeats

```
>Bottlenose_dolphin_IVL2
MSQQCTLPVTLPPAPSKEPLKPVSPPTNIQQ
EQVKQPTPLPAPSQKVPSEIPGKDPMEFGKK
HTTPVKEVPKKECEP
RQQEP-QQQEQQQQQQQQQQQQQQK
-SEEQGKHVEQQQQQQ----------
ESQEQGKPVEQQQQ----------KK
VSQEQGKHVEQQQQQ----------K
ESQEQGKPVEQQQQQQQSHRRKESL
```
rep.

```
>Vaquita_IVL2
MSQQLTLPVTLPPAPSKESLKPVSPPTNIQQ
EQAKQPTPLPAPFQKVSSEFPGKDPMEVGKK
HTTPVKEVPKKECEP
RQQEPQQQQQQQQQQ-
ESQKPGKPVDQQQQQ-K
ESQEPGKPVDQQQQQ-K
ESQEPGKPVDQQQQQ-K
ESQEPGKPVDQQQQQ-K
ESQKPGKHVQQQQQQ-K
ESQEPGKHVEQQQQQ-K
ESQEPGKPVDQQQQQ-K
ESQKPGKPVDQQQQQ-K
ESQKPGKPVDQQQQQ-K
ESQEPGKPVDQQQQQ-K
ESQEPGKPVDQQQQQQK
ESQEPGKPVDQQQQQ-K
ESQEPGKPVDQQQQQ-K
ESQEPGKPVDQQQQQ-K
ESQEPGKPVDQQQQQ-K
ESQEPGKHVDQQQQQ-K
ESQEPGKHVQQLKQE-KKVLGQRLDQELA
```
repeats

Supplementary Figure S5. Sequence repeats of involucrin (IVL) proteins in cattle and cetaceans. Amino acid sequences were aligned to highlight the internal sequence repeats. IVL sequences of bottlenose dolphin (*Tursiops truncatus),* vaquita/porpoise (*Phocoena sinus*) and minke whale (*Balaenoptera acutorostrata scammoni*) are encoded by genes listed in Supplementary Tables S2-S4. IVL of cattle (*Bos taurus)* has the GenBank accession number XP_005203889.1. Amino acid residues cysteine (C), proline (P), glutamine (Q), glutamic acid (E) and lysine (K) are highlighted. Sequences were aligned with the MultAlin program (Corpet 1988) and manually adjusted. Dashes were inserted to optimize alignment of sequence repeats. Note that the intra-species similarity of IVL1 and IVL2 is more pronounced than the similarities of IVL1 and IVL2 sequences of different species, suggesting independent IVL gene duplications in the lineages leading to dolphin and vaquita.

```
    Dolphin  MSSQQTG---KDFSKGSSQD---TKGPCPTPAPDTSS----CCGSG-----CCR---DSGCCG-----------CSSVGCCCLPLRRRLLGSRCCCCCCTDSSQR-SRSS---SSDCCGGC
   Porpoise  MSSQQSG---KDFSKGSSQD---TKGPCPTPAPDTSS----CCGSG-----CCG---DSGCCG-----------SSSVGCCCLPLRRRLLGSR-CCCCCADSSQR-SRSS---SSDCCGGC
Minke whale  MTSQQSG---KDFSKGSSQD---AKSSCPTPVPDASS----CCGLG-----CCG---DSGCCG-----------VSSAACCCFPVRRRLQGSRGCCCCCADSSQ-SSGSS---SSGCCGAC
     Cattle  MSSQQSA---KGSPRGSSQGPTPCPAPCPAPEPDTSSCSSGCCGNGCCGNGCCGSSGDSGCCGDCGCGDCGCGGSSSVGCCCFPRRRRRQGRR-GCGCCGGSSQRSQYSS---SSGCC-GC
      Human  MSSQQSAVSAKGFSKGSSQGPAPCPAPAPTPAP-ASS-SS-CCGSG---RGCCG---DSGCCG-----------SSSTSCCCFPRRRRRQ-RSSGCCCCGGGSQRSQRS-NNRSSGCCSGC
```

Supplementary Figure S6. Cysteine-rich C-terminal protein 1 (CRCT1) is conserved in cetaceans. Amino acid sequence alignment of the cysteine-rich C-terminal protein 1 (CRCT1) of dolphin, porpoise and whale with cattle and human. CRCT1 sequences of dolphin (*Tursiops truncatus),* porpoise (*Phocoena sinus*) and minke whale (*Balaenoptera acutorostrata scammoni*) derived from genes listed in Supplementary Tables S2-S4. CRCT1 of cattle (*Bos taurus*) (accession number NP_001070988.1) and human (*Homo sapiens*) (accession number NP_061933.1) are from GenBank. Amino acid residues cysteine (C), proline (P), glutamine (Q) and lysine (K) are highlighted. Sequences were aligned with the MultAlin program (Corpet 1988) and manually adjusted.

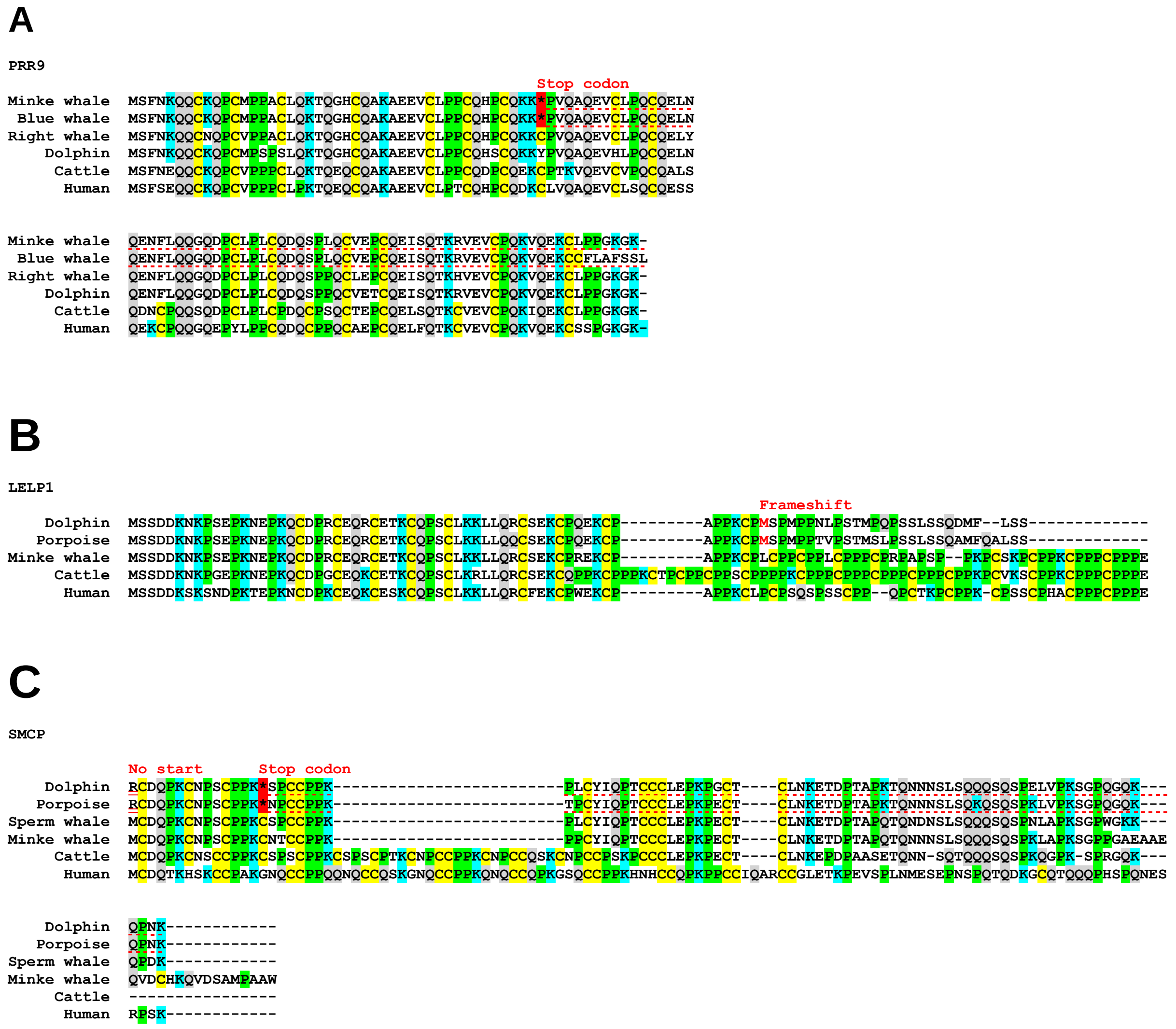

Supplementary Figure S7. Differential conservation of PRR9, LELP1 and SMCP in cetaceans. (A) Amino acid sequence alignment of PRR9 of minke, blue and right whale and dolphin with cattle and human. Among baleen whales investigated (Supplementary Table S1, S4, S5), only the right whale has an intact *PRR9* gene. *PRR9* was not found in the porpoise. Sequences used: dolphin XP_004331701.1, minke whale XP_007178638.1, cattle XP_002686070.2, human NP_001182500.1. **(B)** Amino acid sequence alignment of LELP1 of dolphin, porpoise, minke whale, cattle and human. Sequences used: minke whale NP_001070524.1, cattle XP_002686070.2 and human NP_001010857.1. The LELP1 sequences of dolphin and porpoise differed from the database prediction (Supplementary Tables S2 and S3). **(C)** Amino acid sequence alignment of SMCP of dolphin, porpoise, sperm whale, minke whale, cattle and human. Sequences used: cattle NP_001008417.2 and human NP_109588.2. SMCP of minke whale (Supplementary Table S4). Amino acid residues cysteine (C), proline (P), glutamine (Q) and lysine (K) are highlighted. The symbol "*", highlighted in red, marks a premature stop codon. Amino acid sequences derived by translation of nucleotides after stop codons are underlined with a broken line. Species: dolphin (*Tursiops truncatus*), porpoise (*Phocoena sinus*), sperm whale (*Physeter catodon*), minke whale (*Balaenoptera acutorostrata scammoni*), blue whale (*Balaenoptera musculus*), North Pacific right whale (*Eubalaena japonica*), cattle (*Bos taurus*) and human (*Homo sapiens)*. Sequences were aligned with the MultAlin program (Corpet 1988) and manually adjusted.

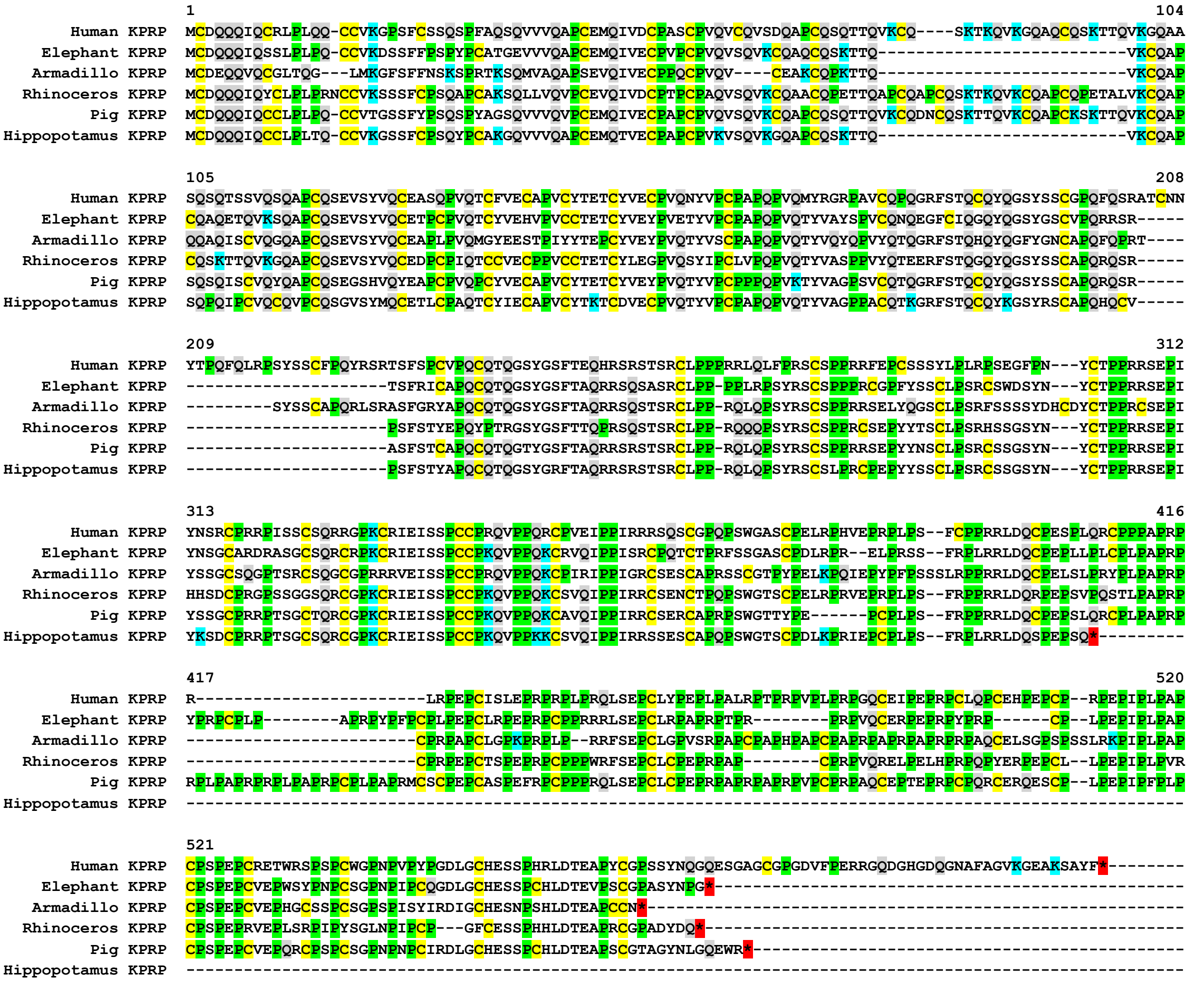

Supplementary Figure S8. Amino acid sequence alignment of keratinocyte proline rich proteins (KPRPs). Amino acid sequences of KPRPs of phylogenetically diverse species of mammals were aligned. To indicate the peculiar amino acid composition of KPRPs, the following amino acid residues are highlighted: lysine (K) and glutamine (Q) are potential sites of transglutamination; cysteine residues (C) are potential sites of disulfide bonds; proline (P) is highly abundant in KPRP. An asterisk on red background indicates the carboxy-terminus of each protein. Proteins and GenBank accession numbers: Human KPRP (NP_001020402.1), elephant KPRP (XP_010587944), armadillo KPRP (XP_004469800), rhinoceros KPRP (XP_004444134), pig KPRP (XP_013852561.2), hippopotamus KPRP (translation of nucleotide sequence, accession number PVJP02004962.1, nucleotides 460180- 461223).

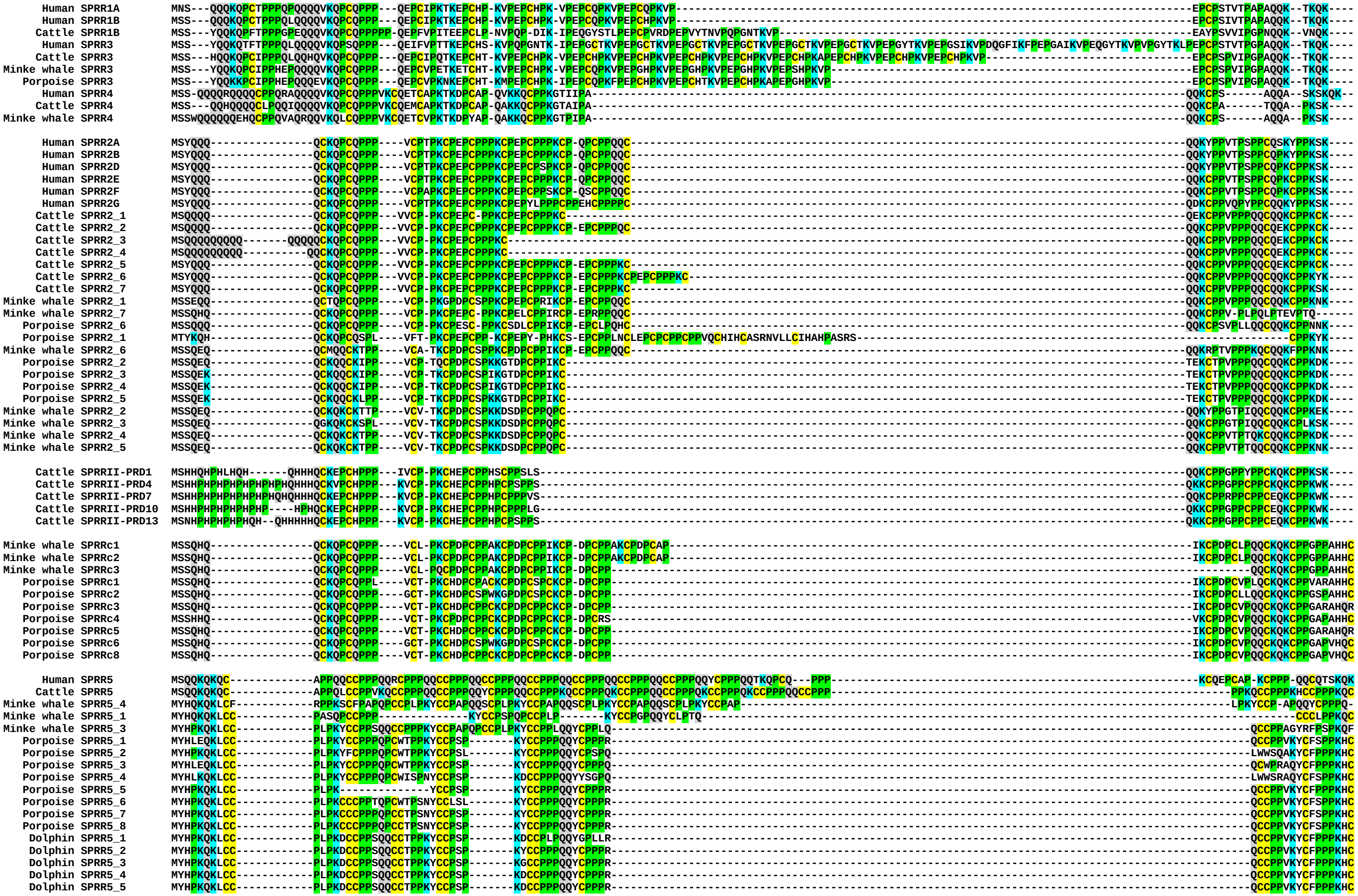

Supplementary Figure S9. Amino acid sequence alignment of SPRRs of cetaceans, cattle and human highlighting sequence conservation within subtypes and differences between subtypes. Small proline rich protein (SPRR) sub-types are separated by a blank line. Note that not all SPRRs of each species are included. Species: Cattle (*Bos taurus*), dolphin (*Tursiops truncatus*), human (*Homo sapiens*), minke whale (*Balaenoptera acutorostrata scammoni*), porpoise (vaquita, *Phocoena sinus*).

A

Bottlenose dolphin (*Tursiops truncatus*) SPRR gene cluster on chromosome 10 (uncertain)

Gene	Accession nr.	start	end	Notes
KHDRBS2	NC_047043.1	45040789	45730010	
n.a.	NC_047043.1	45866722	45903412	sequence gap
SPRR5-like_1	NC_047043.1	45903553	<45903413	partial due to gap in the database
SPRR5-like_2	NC_047043.1	45912755	45912558	sequence identical to SPRR5_4 and SPRR5_5 in EDC
SPRR5-like_3	NC_047043.1	45921967	45921770	sequence identical to SPRR5_4 and SPRR5_5 in EDC
SPRR5-like_4	NC_047043.1	45930969	45930772	
SPRR5-like_5	NC_047043.1	45939980	45939783	
SPRR5-like_6	NC_047043.1	45948979	45948782	sequence identical to SPRR5_4 and SPRR5_5 in EDC
SPRR5-like_7	NC_047043.1	45958182	45957985	sequence identical to SPRR5_4 and SPRR5_5 in EDC
SPRR5-like_8	NC_047043.1	45967386	45967189	
SPRR5-like_9	NC_047043.1	45976540	45976343	sequence identical to SPRR5_4 and SPRR5_5 in EDC
n.a.	NC_047043.1	45984687	45985188	sequence gap
NEK10	NC_047043.1	46388776	46078997	

B

>Tt_SPRR5-like_1_partial
MYHPKQKLSARYQVLLPSIPAMLHPTKYCCPSPKYCCPPPQQYCPLPXXX

>Tt_SPRR5-like_2
MYHPKQKLCCPLPKDCCPPSQQCCTPPKYCCPSPKDCCPPPQQYCPPPRQCCPPVKYCFPPPKHC

>Tt_SPRR5-like_3
MYHPKQKLCCPLPKDCCPPSQQCCTPPKYCCPSPKDCCPPPQQYCPPPRQCCPPVKYCFPPPKHC

>Tt_SPRR5-like_4
MYHPKQKLCCPLPKDCCPPSQQCCTPPKYCCPSPKDCCPPPQQYCPPLRQCCPPVKYCFPPPKHC

>Tt_SPRR5-like_5
MYHPKQKLCCPLPKDCCPPSQQCCTPPKYCCPSPKDCCPPPQQYCPPLRQCCPPVKYCFPPPKHC

>Tt_SPRR5-like_6
MYHPKQKLCCPLPKDCCPPSQQCCTPPKYCCPSPKDCCPPPQQYCPPPRQCCPPVKYCFPPPKHC

>Tt_SPRR5-like_7
MYHPKQKLCCPLPKDCCPPSQQCCTPPKYCCPSPKDCCPPPQQYCPPPRQCCPPVKYCFPPPKHC

>Tt_SPRR5-like_8
MYHPKAEAVLPATKGLLPPSQQCCTPPKYCCPSPKDCCPPPQQYCPPPRQCCPPVKYCFPPPKHC

>Tt_SPRR5-like_9
MYHPKQKLCCPLPKDCCPPSQQCCTPPKYCCPSPKDCCPPPQQYCPPPRQCCPPVKYCFPPPKHC

Supplementary Figure S10. SPRR5-like genes possibly located outside of the EDC of the bottlenose dolphin (*Tursiops truncatus*). (A) Gene positions. Nucleotide numbers corresponding to the start and end of coding sequences and gaps before and after the SPRR cluster are indicated. Note that these genes are currently (12.02.2021) annotated as non-coding RNAs or not annotated in GenBank. The location on chromosome 10 is considered uncertain. **(B)** Amino acid sequences of proteins encoded by SPRR5-like genes. "XXX" indicates a stretch of unknown amino acid sequence due to a gap in the corresponding gene sequence. n.a., not applicable; nr., number; SPRR, small proline rich protein; Tt, *Tursiops truncatus*.
